# Dabrafenib inhibits the growth of *BRAF‐WT* cancers through CDK16 and NEK9 inhibition

**DOI:** 10.1002/1878-0261.12152

**Published:** 2017-11-23

**Authors:** Manali Phadke, Lily L. Remsing Rix, Inna Smalley, Annamarie T. Bryant, Yunting Luo, Harshani R. Lawrence, Braydon J. Schaible, Yian A. Chen, Uwe Rix, Keiran S. M. Smalley

**Affiliations:** ^1^ The Department of Tumor Biology The Moffitt Cancer Center & Research Institute Tampa FL USA; ^2^ The Department of Drug Discovery The Moffitt Cancer Center & Research Institute Tampa FL USA; ^3^ The Chemical Biology Core The Moffitt Cancer Center & Research Institute Tampa FL USA; ^4^ The Department of Biostatistics and Bioinformatics The Moffitt Cancer Center & Research Institute Tampa FL USA; ^5^ The Department of Cutaneous Oncology The Moffitt Cancer Center & Research Institute Tampa FL USA

**Keywords:** BRAF, CDK16, chemical proteomics, dabrafenib, melanoma, NEK9, NRAS, pancreatic, trametinib

## Abstract

Although the BRAF inhibitors dabrafenib and vemurafenib have both proven successful against *BRAF*‐mutant melanoma, there seem to be differences in their mechanisms of action. Here, we show that dabrafenib is more effective at inhibiting the growth of *NRAS*‐mutant and *KRAS*‐mutant cancer cell lines than vemurafenib. Using mass spectrometry‐based chemical proteomics, we identified NEK9 and CDK16 as unique targets of dabrafenib. Both NEK9 and CDK16 were highly expressed in specimens of advanced melanoma, with high expression of both proteins correlating with a worse overall survival. A role for NEK9 in the growth of *NRAS‐* and *KRAS*‐mutant cell lines was suggested by siRNA studies in which silencing was associated with decreased proliferation, cell cycle arrest associated with increased p21 expression, inhibition of phospho‐CHK1, decreased CDK4 expression, and the initiation of a senescence response. Inhibition of CDK4 but not CHK1 recapitulated the effects of NEK9 silencing, indicating this to be the likely mechanism of growth inhibition. We next turned our attention to CDK16 and found that its knockdown inhibited the phosphorylation of the Rb protein at S780 and increased expression of p27. Both of these effects were phenocopied in *NRAS‐* and *KRAS‐*mutant cancer cells by dabrafenib, but not vemurafenib. Combined silencing of NEK9 and CDK16 was associated with enhanced inhibition of melanoma cell proliferation. In summary, we have identified dabrafenib as a potent inhibitor of NEK9 and CDK16, and our studies suggest that inhibition of these kinases may have activity against cancers that do not harbor *BRAF* mutations.

**Abbreviations**
ALK5: transforming growth factor receptor beta 1
ATP: adenosine triphosphate
BRAF: B‐Raf proto‐oncogene, serine/threonine kinase
CAMK1: calmodulin kinase 1
CDK16: cyclin‐dependent kinase‐16
CDK4: cyclin‐dependent kinase‐4
CHK1: checkpoint kinase 1
CML: chronic myeloid leukemia
CRAF: V‐Raf‐1 murine leukemia viral oncogene‐like protein 1
FDA: Food and Drug Administration
FGR: Gardner‐Rasheed feline sarcoma viral oncogene homolog
GADD45A: growth arrest and DNA damage‐inducible alpha
HRP: horseradish peroxidase
MAPK: mitogen‐activated protein kinase
NEK9: never in mitosis gene A‐9
NSCLC: non‐small‐cell lung cancer
OS: overall survival
PFS: progression‐free survival
PI3K: phosphoinositide 3‐kinase
q‐RT‐PCR: quantitative reverse transcription polymerase chain reaction
RB: retinoblastoma protein
RTK: receptor tyrosine kinase
SCC: squamous cell carcinoma
SIK2: salt‐inducible kinase 2
siRNA: short interfering ribonucleic acid
SRMS: Src‐related kinase lacking C‐terminal regulatory tyrosine and N‐terminal myristoylation sites
TCGA: The Cancer Genome Atlas

## 1 Introduction

The development of small‐molecule BRAF inhibitors and the BRAF‐MEK inhibitor combination has revolutionized the treatment for *BRAF*‐mutant melanoma. At this time, two BRAF inhibitors have been FDA‐approved for the treatment of advanced (stage IV) unresectable melanoma (Flaherty *et al*., 2010, 2012). The first to be developed was vemurafenib (originally named PLX4032), which received FDA approval in 2011. This followed the completion of a successful randomized phase II trial in which it outperformed the previous standard‐of‐care dacarbazine (Flaherty *et al*., 2010). Side effects to vemurafenib were generally mild and included nausea, vomiting, rash, and fatigue (Chapman *et al*., 2011). One unexpected off‐target effect of vemurafenib was the rapid development of cutaneous lesions including squamous cell carcinomas (SCC) (frequently of the rare keratoacanthoma type), actinic keratoses, and secondary (non‐*BRAF*‐mutant) melanomas (Gibney *et al*., 2013; Su *et al*., 2012). Mechanistic studies revealed the development of these secondary tumors to be a consequence of BRAF inhibitors transactivating CRAF in pre‐existing subclinical lesions that harbored mutations in *HRAS* (Poulikakos *et al*., 2010; Su *et al*., 2012). This phenomenon, which is commonly referred to as paradoxical mitogen‐activated protein kinase (MAPK) pathway activation, is known to occur when BRAF inhibitors are applied to cells with other upstream activators of the MAPK pathway including *NRAS* and *KRAS* mutations and high levels of receptor tyrosine kinase (RTK) amplification/signaling (Poulikakos *et al*., 2010).

Dabrafenib was developed after vemurafenib, receiving FDA approval in 2013. As a single agent, the progression‐free survival (PFS) of patients on dabrafenib therapy was similar to that of vemurafenib. Despite single‐agent vemurafenib and dabrafenib having a common mechanism of action (inhibition of mutant BRAF) and mostly similar side‐effect profiles, some differences were noted in the mechanisms of therapeutic escape between the two drugs. Acquired resistance to both vemurafenib and dabrafenib is most often associated with recovery of signaling through the MAPK pathway resulting from acquired mutations in *MEK1/2* mutations, *BRAF*‐splice mutants, and adaptive RTK signaling (Nazarian *et al*., 2010; Poulikakos *et al*., 2011; Shi *et al*., 2014a,2014b). Genetic lesions in the PTEN/phosphoinositide 3‐kinase (PI3K) and AKT signaling pathway have also been commonly reported in up to 22% of cases, both alone and concurrently with MAPK pathway alterations (Shi *et al*., 2014a,2014b). Of note, acquired *NRAS* mutations occurred more frequently in patients failing vemurafenib compared to dabrafenib ([OR] 3.53, *P* = 0.045) (Johnson *et al*., 2015). One explanation for these clinical observations was potential differences in the off‐target kinase activity of the two BRAF inhibitors. To better understand these observations, we used an unbiased chemical proteomic screen to identify kinases that were unique to vemurafenib and to dabrafenib. Our studies identified NEK9 and CDK16 as two dabrafenib‐specific kinase targets and provide evidence that inhibition of NEK9 and CDK16 may underlie the greater effectiveness of this drug against *NRAS‐* and *KRAS*‐mutant cancer cells.

## 2 Materials and methods

### 2.1 Cell culture

The parental *BRAF‐*mutant melanoma cell line 1205Lu and *NRAS*‐mutant melanoma cell line WM1366 were a kind gift from Meenhard Herlyn (The Wistar Institute, Philadelphia, PA). The other melanoma cell lines M245, M249, M249R were provided by Antoni Ribas (UCLA, Los Angeles, CA), and IPC‐298 was purchased from the Leibniz‐Institut DSMZ (Germany). The *KRAS*‐mutant pancreatic cell lines CAPAN‐1 and MIA PaCa‐2 were provided by Shari A. Pilon‐Thomas (H. Lee Moffitt Cancer Center, Tampa, FL). The identity of each cell line was confirmed through STR validation performed by Biosynthesis Inc. (Lewisville, TX). All the cell lines were cultured in RPMI complete medium supplemented with 5% fetal bovine serum (FBS). The acquired resistant cell lines were cultured in RPMI complete medium with 5% FBS with the addition of 2 μm vemurafenib.

### 2.2 Cell proliferation assay

Cells were grown overnight at a density of 5 × 10^4^ cells per mL and were treated with vehicle (dimethyl sulfoxide, DMSO), 100 nm dabrafenib (Selleckchem), and 1 μm vemurafenib (Selleckchem) or were transfected with siRNA overnight. Cells were counted either for 7 days after drug treatment or for 5 days after siRNA transfection. The cells were counted daily using trypan blue. The percentage of total cells was normalized to the percentage of control cells.

### 2.3 Colony formation assay

Cells were grown overnight at a density of 1 × 10^4^ cells per mL and treated with vehicle (DMSO), 100 nm, 1 μm of vemurafenib, or with 100 nm of dabrafenib. The medium and drug/vehicle was replaced every two weeks. After 4 weeks of treatment, colonies were stained with crystal violet dye, as described in Paraiso *et al*. (2010). The percentage relative clonogenic survival was determined by dissolving the crystal violet dye in 10% acetic acid solution. The absorbance was read at 450 nm.

### 2.4 Flow cytometry for apoptosis analysis

Cells were plated into 6‐well plates at a density of 1 × 10^5^ cells per mL (at about 30% confluency) and left to adhere overnight. Cells were treated with 1 μm of vemurafenib or with 100 nm of dabrafenib for 48 h or transfected with siRNA overnight. Annexin V staining quantification was performed using FlowJo software as described in Paraiso *et al*. (2012).

### 2.5 Synthesis of vemurafenib, dabrafenib, and trametinib analogues

See Supplemental Methods in Appendix S1 for chemical synthesis.

### 2.6 Chemical proteomics

Cells were harvested, pelleted by centrifugation, and lysed with an equal volume of lysis buffer as described previously (Rix *et al*., 2007). Lysates were centrifuged twice at 21 000 ***g*** and 4 °C (10 min, 20 min), and the protein concentration was determined using a Bradford assay. Drug affinity experiments were performed in duplicate essentially as described before (Rix *et al*., 2007) with the difference of using 1 mg of protein per pulldown for samples intended for mass spectrometry and 5 mg protein per pulldown for samples intended for immunoblot analysis. Competition was performed by cotreatment of total cell lysates with 20 μm of the respective unmodified inhibitors during incubation with drug affinity matrices. SDS/PAGE, in‐gel digestion with trypsin, and LC‐MS/MS analyses were performed as described previously (Wright *et al*., 2017). Data were searched against the SwissProt 2014_08 (1205Lu cells) and 2015_12 (WM1366 cells) human protein database using the Sequest (1205Lu cells) and Mascot (WM1366 cells) search engines. Results were visualized in Scaffold (www.proteomesoftware.com). Relative protein quantification was performed using normalized spectral abundance factors (NSAF) (Zybailov *et al*., 2007).

### 2.7 Western blotting

Protein extraction and western blotting were performed as per the methods in Smalley *et al*. (2007). The primary antibodies for phospho‐ERK, total‐ERK, phospho‐Chk1, p21, γ‐H2AX, phospho‐Rb (Ser 780), and p27 were from Cell Signaling Technology. The Nek9 antibody was from Abcam, the Cdk16 antibody from Santa Cruz Biotechnology, and the glyceraldehyde‐3‐phosphate dehydrogenase (GAPDH) antibody from Sigma Aldrich. The secondary antibodies goat anti‐rabbit IgG HRP and sheep anti‐mouse IgG HRP were from Amersham/GE Healthcare.

### 2.8 RNA interference

1205Lu and WM1366 cells were plated at a cell density of 1 × 10^5^ cells per mL and left overnight to grow in complete RPMI medium with 5% FBS. The complete medium was replaced with Opti‐MEM (Invitrogen). Cells were transfected with siRNA # 1 for NEK9 (Santa Cruz Biotechnology; Cat no. sc‐61178), siRNA # 2 for Nek9 (Dharmacon SMARTpool; Cat no. L‐004869‐00‐0005) and CDK16 (Ambion; Cat no. AM51331) in complex with Lipofectamine 2000 (Invitrogen, Cat no. 11668019). 50 nm of siRNA concentration was used, and cells were transfected overnight. Nontargeting siRNAs (Santa Cruz; Cat no. sc‐37007) were added as an siRNA control. Following the transfection time, cells were replenished with complete RPMI medium with 5% FBS and treated with 1 μm vemurafenib or 100 nm dabrafenib for 48 h.

### 2.9 Flow cytometry for cell cycle analysis

Cells were plated into 6‐well plates at a density of 1 × 10^5^ cells per mL and left to adhere overnight. They were then treated with 1 μm of vemurafenib or with 100 nm of dabrafenib for 48 h or transfected with siRNA overnight. The cells were trypsinized and fixed overnight in 70% ice‐cold ethanol. Propidium iodide was used to stain the cells, and cell cycle analysis was performed by flow cytometry using Canto and ModFit software.

### 2.10 TCGA analysis

The level‐3 RNA‐Seq expression data and clinical covariates from TCGA melanoma patient samples were downloaded through cBioportal (*N* = 338 unique patients). Overall survival (OS) is defined from the time of sample collection to death and censored at the last follow‐up. The optimal cut‐point for RNA‐Seq expression for NEK9 or CDK16 for survival analyses was chosen using the maximally selected rank statistic in the ‘coin’ R package. Both the unadjusted *P*‐values of log‐rank tests and the *P*‐values penalized by multiple looks are reported. The results for survival analyses using the identified optimal cut‐point for NEK9 and CDK16 were visualized using Kaplan–Meier curves. For the subgroup of patients with *BRAF‐* mutations (*N* = 151), we performed the log‐rank test using the cut‐point defined from the overall analysis; therefore, no penalty was imposed.

## 3 Results

### 3.1 Dabrafenib inhibits the growth of *RAS*‐mutant cell lines

**Figure 1:**
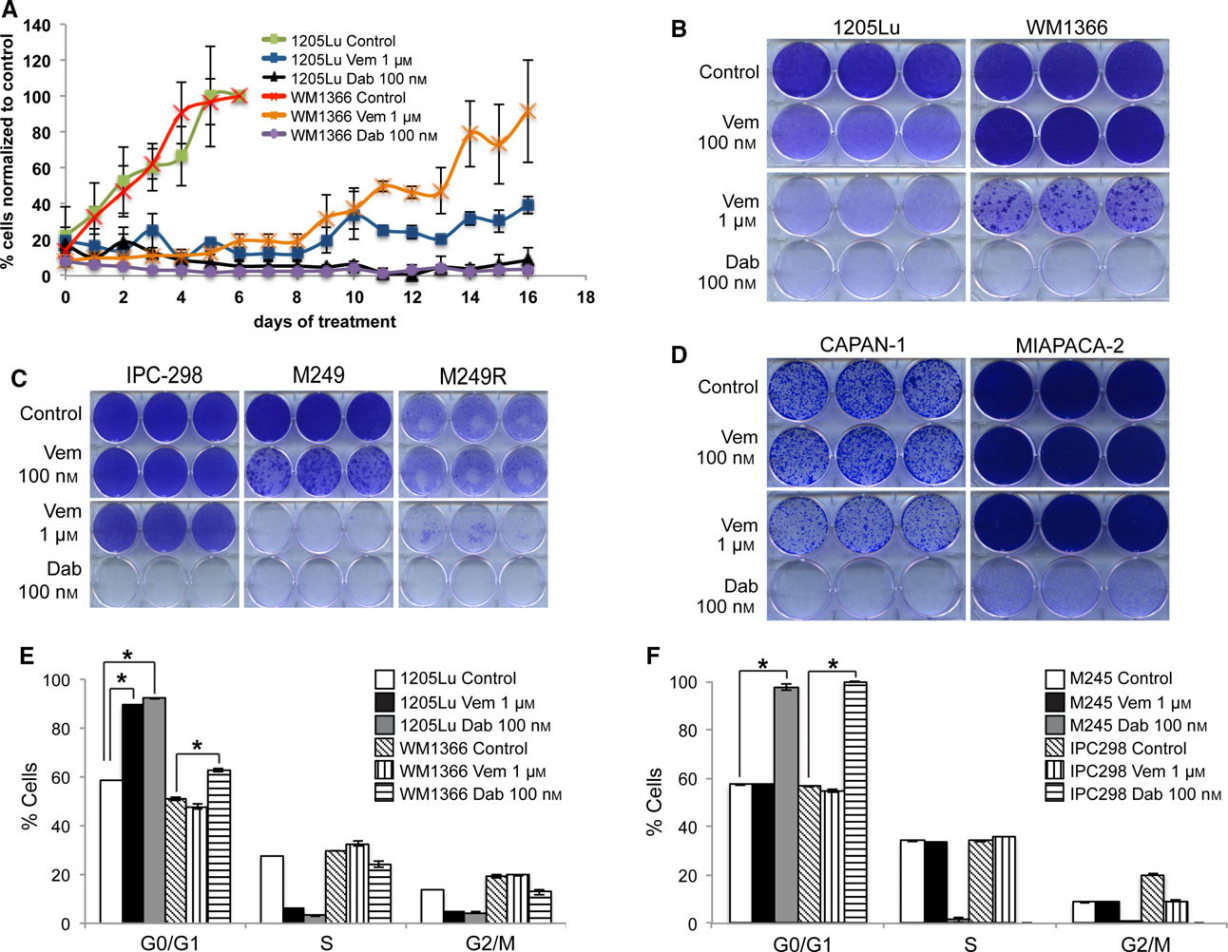
Growth inhibition of *BRAF*‐ and *RAS*‐mutant cancer cell lines by dabrafenib and vemurafenib. (A) long‐term growth assays of *NRAS*‐mutant (WM1366) and *BRAF*‐mutant melanoma (1205Lu) cells treated with either vemurafenib or dabrafenib for 16 days. Cells were counted daily and expressed as a % of control cell growth. (B) long‐term colony formation assays of 1205Lu and WM1366 cells treated with either vemurafenib or dabrafenib for 4 weeks. Cells were visualized by staining with crystal violet. (C) long‐term colony formation assays of *NRAS‐*mutant (IPC‐298, M249R) and *BRAF*‐mutant melanoma cells (M249) cells treated with either vemurafenib or dabrafenib for 4 weeks. Cells were visualized by staining with crystal violet. (D) long‐term colony formation assays of *KRAS*‐mutant pancreatic cancer cell lines (CAPAN‐1 and MIA PaCa‐2) treated with either vemurafenib or dabrafenib for 4 weeks. Cells were visualized by staining with crystal violet. (E) cell cycle analysis of 1205Lu and WM1366 cells treated with either vemurafenib or dabrafenib for 48 hrs. Cells were stained with propidium iodide and analyzed by flow cytometry. (F) cell cycle analysis of M245 and IPC‐298 cells treated with either vemurafenib or dabrafenib for 48 hrs. Cells were stained with propidium iodide and analyzed by flow cytometry. * indicates *P* < 0.05 compared to control.

As dabrafenib and vemurafenib have different potencies against mutant *BRAF* in isolated kinase assays, we determined equipotent concentrations of drug required to inhibit pERK in 1205Lu *BRAF*‐mutant melanoma cells (Bollag *et al*., 2010; King *et al*., 2013) and identified 100 nm dabrafenib to have equivalent effects to 1 μm of vemurafenib (Fig. S1). To determine whether vemurafenib and dabrafenib had differential effects upon the growth of *BRAF‐* and *NRAS*‐mutant melanoma cells, we treated 1205Lu (*BRAF* mutant) and WM1366 cells (*NRAS* mutant) chronically (>14 days) with each drug and performed cell counts (Fig. 1A). It was found that both BRAF inhibitors suppressed the growth of the 1205Lu cells over 14 days, with little regrowth observed. Treatment of the *NRAS*‐mutant melanoma cells showed a very different pattern of response in which vemurafenib initially suppressed growth, followed by escape and regrowth. In contrast, dabrafenib treatment led to a long‐term suppression of growth, and no escape (Fig. 1A). The durability of these responses was determined by 4‐week colony formation assays (Fig. 1B) in which dabrafenib completely suppressed the WM1366 cell growth, but vemurafenib did not. In *BRAF*‐mutant 1205Lu and M249 cells, both dabrafenib and vemurafenib had similar efficacy at suppressing long‐term growth (Fig. 1B, 1C). These effects were not confined to the WM1366 cell line as dabrafenib also exhibited long‐term growth suppression of other *RAS*‐mutant melanoma cancer cell lines, including IPC‐298 (*NRAS* mutant) and M249R cells (a cell line that was initially *BRAF* mutant and acquired an *NRAS* mutation upon BRAF inhibitor resistance) (Nazarian *et al*., 2010)(Fig. 1C) and two *KRAS*‐mutant pancreatic cancer cell lines (Capan‐1 and MIA PaCa‐2) (Fig. 1D). Functionally, dabrafenib led to a growth arrest in the *BRAF‐* and *RAS*‐mutant melanoma cell lines, with a G1‐phase cell cycle arrest noted (Fig. 1E and 1F) (~ 10% in WM1366 cells). Vemurafenib was only associated with G1‐phase cell cycle arrest in the *BRAF*‐mutant melanoma cells and not those harboring *NRAS* mutations (Fig. 1E & F).

### 3.2 Chemical proteomic analysis shows dabrafenib and vemurafenib to interact with different kinase targets

**Figure 2:**
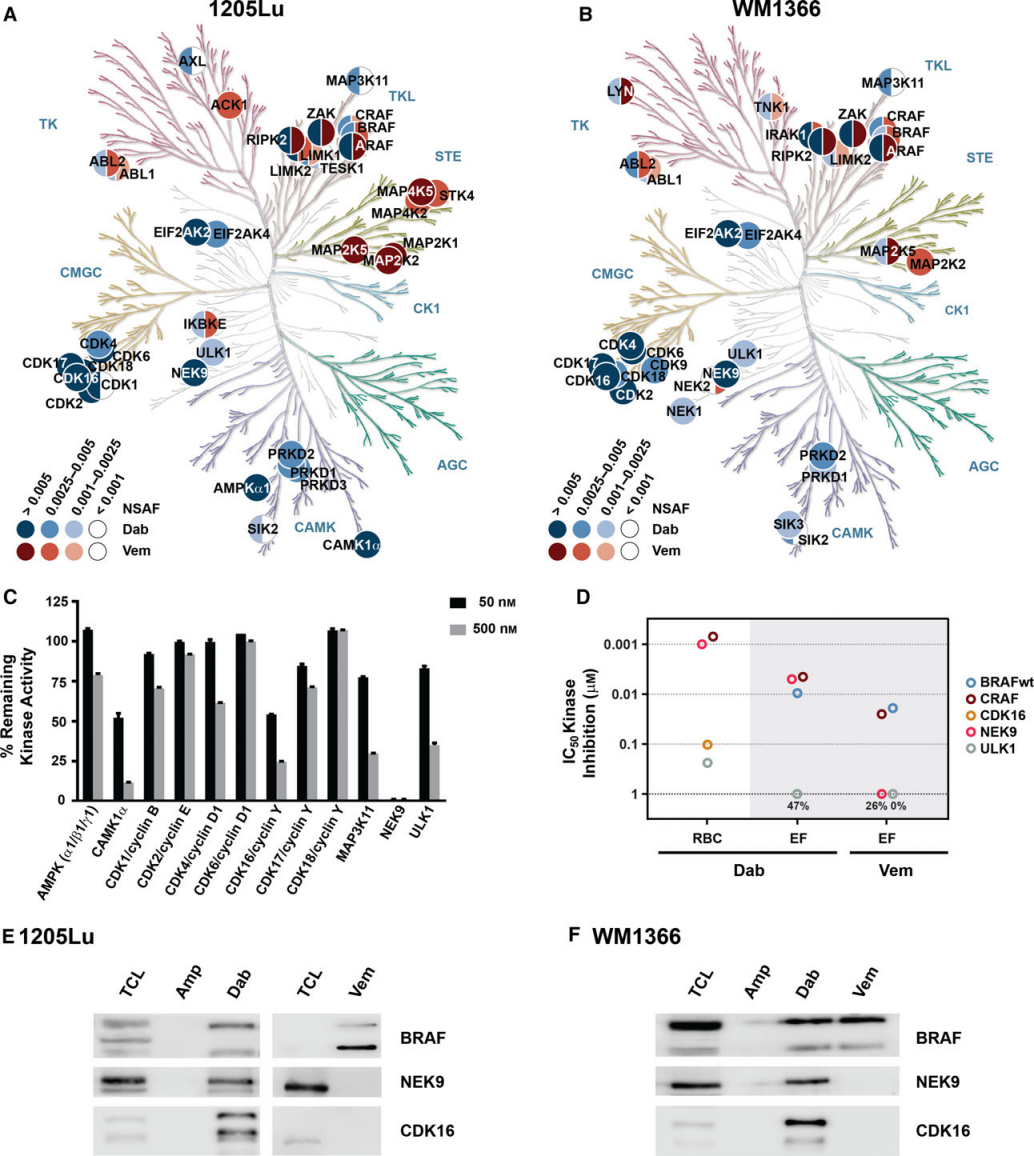
Mass spectrometry‐based chemical proteomics analysis of dabrafenib and vemurafenib target profiles in melanoma cells. (A) kinome tree of potential kinase targets of vemurafenib (red) and dabrafenib (blue) in *BRAF*‐mutant 1205Lu melanoma cells. Values given are the normalized spectral abundance factor (NSAF). (B) kinome tree of potential kinase targets of vemurafenib (red) and dabrafenib (blue) in *NRAS*‐mutant WM1366 melanoma cells. Values given are the normalized spectral abundance factor (NSAF). (C) inhibition of multiple kinases by dabrafenib using *in vitro* kinase assays. (D) comparison of vemurafenib and dabrafenib IC_50_ values for the inhibition of wild‐type BRAF, CRAF, CDK16 (not available from EF), NEK9, and ULK1 from two providers (EF: Eurofins, RBC: Reaction Biology Corp). % values indicate % kinase inhibition at 1 μm drug. (E) Dabrafenib binds BRAF, NEK9, and CDK16, whereas vemurafenib only binds BRAF in 1205Lu lysate. Immobilized ampicillin was used as negative control. (F) Dabrafenib binds BRAF, NEK9, and CDK16 in WM1366 lysates, whereas vemurafenib only binds BRAF. Immobilized ampicillin was used as negative control.

As dabrafenib and vemurafenib displayed different activity against *BRAF‐* and *RAS‐*mutant melanoma cell lines, we next asked whether dabrafenib had unique kinase targets. To explore this, we applied an unbiased chemical proteomics approach (Rix and Superti‐Furga, 2009). We synthesized dabrafenib and vemurafenib analogues, named i‐dabrafenib and i‐vemurafenib, which can be immobilized on Sepharose beads (structures shown in Fig. S2A). As MEK inhibitors are used in combination with BRAF inhibitors, we also synthesized an immobilizable analogue, i‐trametinib, of the MEK inhibitor trametinib for inclusion in our analysis (Fig. S2B). The modified dabrafenib, vemurafenib, and trametinib analogues showed similar pharmacological activity to the parent compounds in isolated BRAF‐V600E and MEK2 kinase assays, respectively (Fig. S3). The drug affinity matrices were then incubated with lysates from *BRAF*‐mutant 1205Lu cells or *NRAS*‐mutant WM1366 cells. Subsequent tandem mass spectrometry analysis of drug affinity eluates and relative, label‐free quantification using normalized spectral abundance factors (NSAF) (Zybailov *et al*., 2007) revealed both dabrafenib and vemurafenib to have the expected high affinity for BRAF and CRAF (Fig. 2A,B). However, significant differences in the dabrafenib and vemurafenib target profiles were also noted, with dabrafenib prominently engaging multiple other kinases (Fig. 2A,B). The results from the chemical proteomic screen were validated using *in vitro* kinase assays, which confirmed that dabrafenib significantly inhibits a number of kinases including CAMK1α, MAP3K11, CDK16, and NEK9 (Fig. 2C). Among these, NEK9 was the most potently inhibited and unique dabrafenib target with an IC_50_ value of 1‐9 nm (Fig. 2C,D) followed by CAMK1α and CDK16 (Fig. 2C). As CAMK1α was not expressed in WM1366 cells (data not shown), we focused on the roles of NEK9 and CDK16 as the most potent new dabrafenib targets in WM1366 cells. The targeting of NEK9 and CDK16 by dabrafenib was validated by western blot of 1205Lu and WM1366 cell lysates in which i‐dabrafenib and i‐vemurafenib both pulled down BRAF, but only i‐dabrafenib interacted with NEK9 and CDK16 (Fig. 2E,F). In contrast to the multiple kinase targets that interacted with dabrafenib and vemurafenib, the MEK inhibitor trametinib was highly specific, with the canonical targets MEK1 and MEK2 being the most significant interactors (Fig. S4).

### 3.3 NEK9 and CDK16 expression correlates with worse overall survival

**Figure 3:**
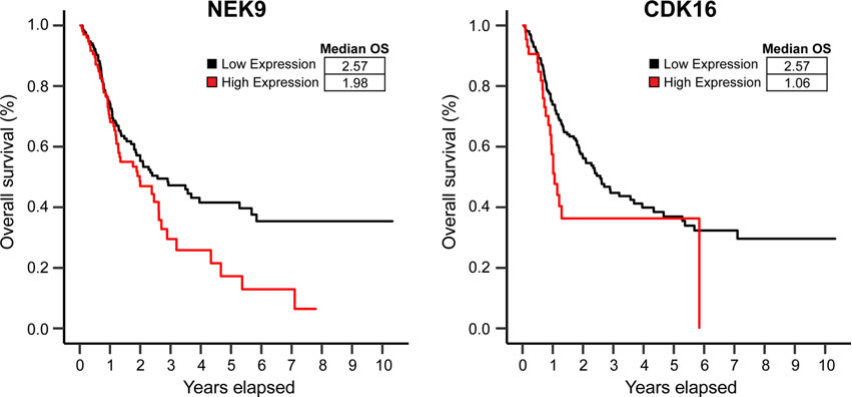
Correlation of NEK9 and CDK16 expression with overall survival in patients with advanced melanoma. (Left) Analysis of the melanoma TCGA dataset showing the correlation between NEK9 expression levels and overall survival. (Right) Analysis of the melanoma TCGA dataset showing the correlation between CDK16 expression levels and overall survival.

Although the role of CDK16 has been explored previously in melanoma progression, little is known about how NEK9 expression impacts long‐term survival. In the TCGA melanoma cohort, the median overall survival (OS) for the low NEK9 expression group was 2.57 years (95% CI: 1.87, 5.68) compared to a median OS for the high NEK9 expression group of 1.98 years (95% CI: 1.27, 2.70). The unadjusted *P*‐value testing the difference in OS between the two groups (low expression and high expression) was 0.04 (Fig. 3). We next explored the link between CDK16 expression and survival in the TCGA melanoma cohort. The median OS for the low expression group was 2.57 years (95% CI: 1.98, 3.96), and the median OS for the high expression group was 1.06 years (0.92, NA). The unadjusted *P*‐value testing for the difference in OS between the two expression groups was 0.04 (Fig. 3). The adjusted *P*‐values, panelized by multiple looks for identifying optimal cut‐points, were not significant (*P* = 0.40 for NEK9 and *P* = 0.52 for CDK16, respectively).

### 3.4 Suppression of NEK9 inhibits the growth of *RAS*‐mutant cells and modulates the expression of p21 and pCHK1

**Figure 4:**
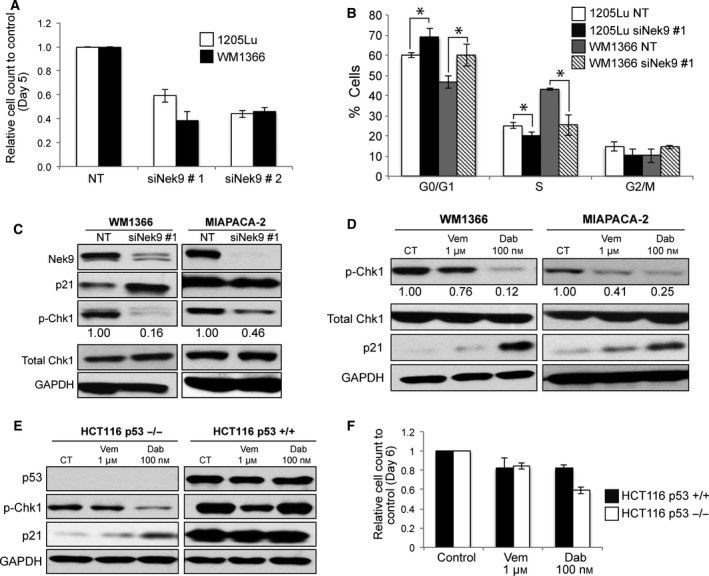
Effect of NEK9 silencing on growth of *RAS*‐mutant cancer cell lines. (A) siRNA knockdown of *NEK9* reduces the growth of *BRAF*‐ and *NRAS*‐mutant melanoma cell lines. Cells were transfected with siRNA #1 or #2 overnight before quantification of cell numbers by trypan blue. (B) *NEK9* silencing (siRNA #1) leads to G1‐phase cell cycle arrest in 1205Lu and WM1366 cells. (C) Knockdown of *NEK9* increases p21 expression and inhibits phosphorylation of CHK1. Cells were transfected with siRNA overnight prior to western blotting for NEK9, p21, pCHK1, CHK1, and GAPDH. Numbers indicate expression relative to control. (D) Dabrafenib, but not vemurafenib, inhibits pCHK1 and increases p21 expression in WM1366 and MIA PaCa‐2 cells. Cells were treated with dabrafenib (100 nm), vemurafenib (1 μm), or vehicle for 24 h (MIA PaCa‐2) or 48 h (WM1366) and subjected to western blot for p‐CHK1, CHK1, p21, and GAPDH. Numbers indicate expression relative to control. (E) Left panel: Dabrafenib selectively inhibits pCHK1 and increases p21 expression in p53−/− cells. HCT116 p53−/− and p53+/+ cells were treated with dabrafenib (100 nm), vemurafenib (1 μm), or vehicle for 48 h and subjected to western blot for p‐CHK1, CHK1, p21, and GAPDH. Right panel: effect of vemurafenib (1 μm) and dabrafenib (100 nm) on proliferation of HCT116 p53−/− and p53+/+ cells. * indicates significant difference from controls (*P* < 0.05).

We next asked whether NEK9 was a functionally relevant dabrafenib target in *RAS*‐mutant cell lines. siRNA‐mediated knockdown of *NEK9* using siRNA pools from two manufacturers (siNEK9#1 and #2) reduced the growth of both *BRAF*‐mutant (1205Lu) and *NRAS*‐mutant (WM1366, IPC‐298, and M245) melanoma cell lines (Fig. 4A and Fig. S5). Silencing of NEK9 did not affect apoptosis (Fig. S6). The effects of *NEK9* knockdown on growth, using two independent NEK9 siRNA pools, were cell cycle dependent and mediated through G1‐phase cell cycle arrest (Fig. 4B and Fig. S7). Mechanistically, the silencing of *NEK9* led to decreased phosphorylation of its downstream substrate CHK1 in WM1366 melanoma cells as well as the MIA PaCa‐2 *KRAS*‐mutant pancreatic cancer cell lines (Fig. 4C) (Kurioka *et al*., 2014). *NEK9* silencing was also associated with increased p21 expression in WM1366 cells. Treatment of WM1366 and MIA PaCa‐2 cells with dabrafenib, but not vemurafenib, was associated with an inhibition of pCHK1 and an increased expression of p21 (Fig. 4D).

Previous work has demonstrated that NEK9 inhibition leads to growth arrest in tumor cell lines that are either null for, or harbor mutations in p53 (Kurioka *et al*., 2014). Among the cell lines used in this study, the WM1366, IPC‐298, MIA PaCa‐2, and CAPAN‐1 harbored p53 mutations, whereas the 1205Lu and M245 were p53 wild‐type (Table S1). To address the role of p53 in the anticancer effects of dabrafenib, we next treated p53+/+ and −/− *KRAS*‐mutant HCT116 cells with drug and found that dabrafenib, but not vemurafenib, induced p21 expression and inhibited pCHK1 in the p53−/− cells (Fig. 4E). In line with this observation, dabrafenib treatment significantly inhibited the growth of the p53−/− HCT116 cells to a greater extent than the isogenic p53+/+ HCT116 cells (Fig. 4F).

### 3.5 NEK9 silencing leads to senescence associated with decreased CDK4 expression

**Figure 5:**
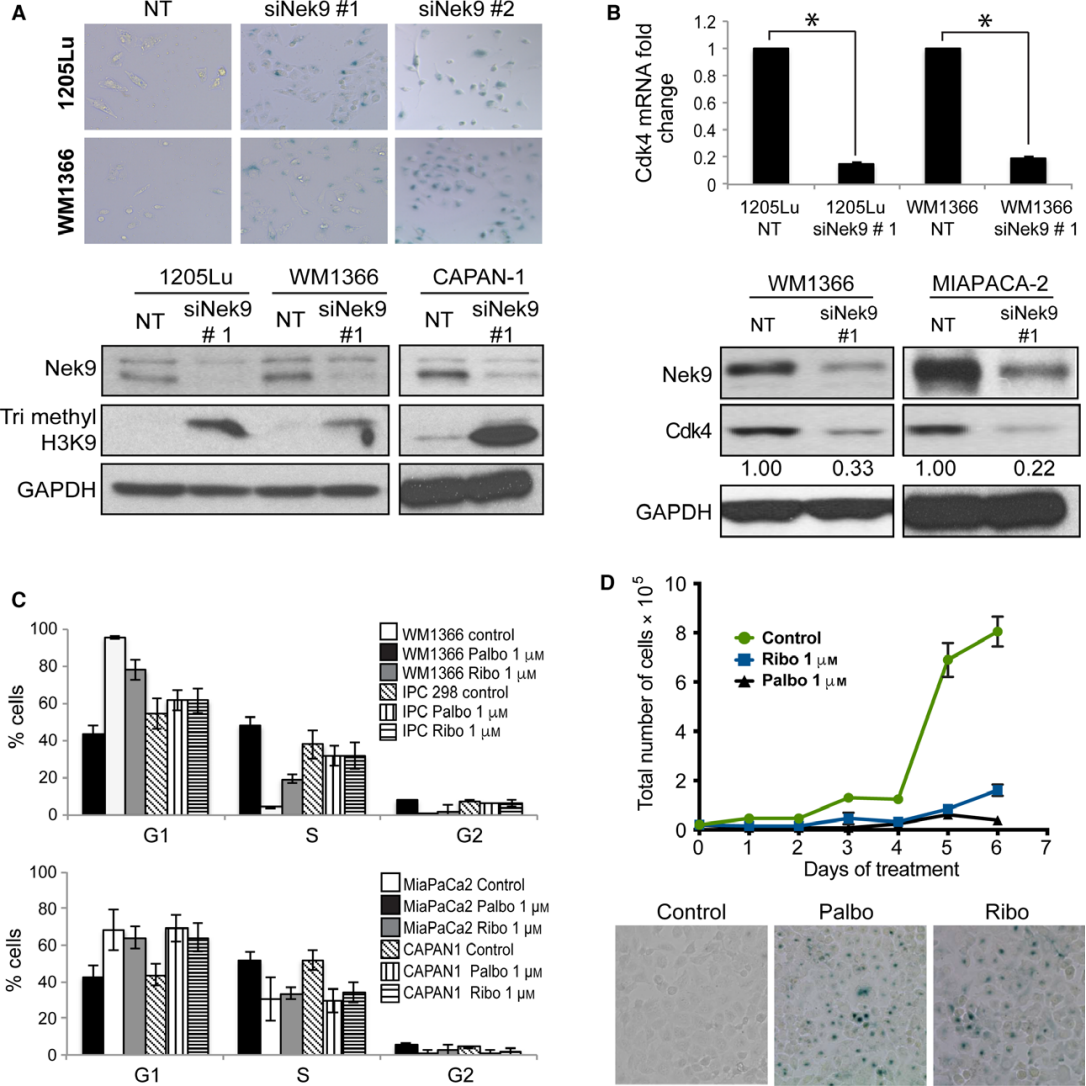
Effect of *NEK9* silencing on CDK4 expression and senescence. (A) siRNA knockdown of *NEK9* leads to senescence induction. Cells were treated with siRNA #1 or #2 overnight before being stained for β‐galactosidase (lower panel). *NEK9* was silenced by siRNA #1 followed by western blotting for trimethylated histone H3K9. (B) *NEK9* silencing leads to decreased CDK4 expression. Upper panel: q‐RT‐PCR for *CDK4* mRNA following *NEK9* silencing. Lower panel: western blot showing that *NEK9* silencing decreases CDK4 expression in both WM1366 and MIA PaCa‐2 cells. Numbers indicate expression relative to control. (C) cell cycle analysis of *NRAS*‐mutant (WM1366 and IPC‐298) melanoma cells and pancreatic cancer cell lines (MIA PaCa‐2 and CAPAN‐1) treated with either palbociclib or ribociclib (both 1 μm) for 48 hrs. Cells were stained with propidium iodide and analyzed by flow cytometry. (D) CDK4 inhibition blocks the growth of WM1366 cells and leads to senescence induction (upper panel). Cells were treated with either palbociclib or ribociclib (both 1 μm) for up to 6 days. Data show mean cell counts (lower panel). β‐Galactosidase staining of WM1366 cells following treatment with either palbociclib or ribociclib (both 1 μm) for 48 h.

Knockdown of NEK9 expression using two siRNA pools (#1 and #2) was associated with an entry into senescence, as demonstrated by increased β‐galactosidase staining and increased expression of senescence‐associated trimethylated histone K9 (Fig. 5A). Previous work has demonstrated NEK9 to regulate expression of mRNAs involved in cell cycle entry, including CDK4 (Kurioka *et al*., 2014). We here show that siRNA knockdown of *NEK9* decreases CDK4 expression at both the mRNA and protein levels (Fig. 5B). A role for CDK4 inhibition in the effects of *NEK9* silencing was suggested by the observation that the CDK4 inhibitors palbociclib and ribociclib induced a G1‐phase cell cycle arrest and led to senescence entry in the WM1366, IPC298, CAPAN‐1, and MIA PaCa‐2 cell lines (Fig. 5C,D and Fig. S8). Although NEK9 directly regulates the phosphorylation of CHK1, inhibition of CHK1 did not appear to mediate the growth arrest and senescence effects of *NEK9* silencing as the CHK1 inhibitor SCH900776 was found to have little effect upon either cell cycle entry or the expression of senescence‐associated β‐galactosidase expression (Fig. S9).

### 3.6 Dabrafenib‐mediated CDK16 inhibition enhances the cell cycle effects of NEK9 inhibition

**Figure 6:**
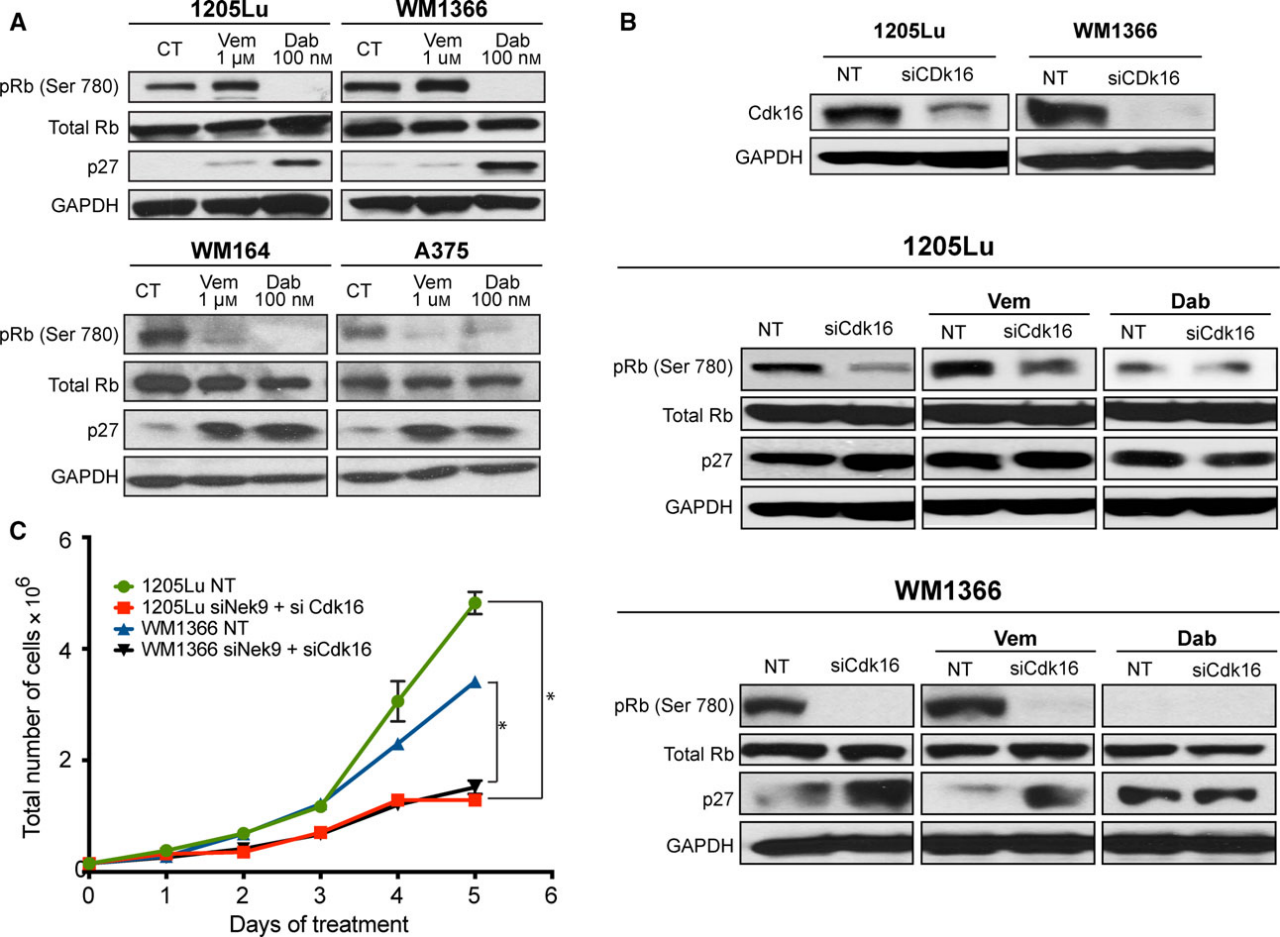
Effect of CDK16 silencing upon the regulation of the cell cycle. (A) (Upper) Dabrafenib, but not vemurafenib, reduces expression of pRB (S780) and increases expression of p27 in 1205Lu and WM1366 cells. (Lower) Both vemurafenib and dabrafenib reduce expression of pRB (S780) and increase expression of p27 in WM164 and WM1366 cells. (B) western blot of pRB (S780) and p27 in 1205Lu and WM1366 cells following knockdown of CDK16 in the absence and presence of vemurafenib (1 μm) or dabrafenib (100 nm). (C) Concurrent knockdown of *NEK9* and *CDK16* leads to a greater inhibition of growth. 1205Lu and WM1366 cells were transfected with *CDK16* and *NEK9* siRNA and cells were counted for 5 days. Data show mean of three experiments ± SEM. * indicates significant difference between siRNA knockdown and control groups (*P* < 0.05).

Dabrafenib inhibits multiple targets in melanoma cells including CDK16. Two major targets of CDK16 are the cell cycle inhibitor p27^KIP1^ and the RB protein (where it mediates its phosphorylation at S780) (Yanagi *et al*., 2014a,2014b). In both the 1205Lu *BRAF*‐mutant melanoma cell line and the WM1366 *NRAS*‐mutant melanoma cell line, treatment with dabrafenib, but not vemurafenib, led to abrogation of RB protein phosphorylation and strong upregulation of p27 (Fig. 6A). In light of the intrinsic resistance to vemurafenib (and its analog PLX4720) shown by the 1205lu *BRAF*‐mutant melanoma cell line (Paraiso *et al*., 2011), we also evaluated vemurafenib and dabrafenib on two highly sensitive *BRAF*‐mutant melanoma cell lines (WM164 and A375) and found both vemurafenib and dabrafenib to be equivalent at inhibiting pRb phosphorylation and inducing p27 expression (Fig. 6A). A role for CDK16 in the regulation of both p27 and pRB was demonstrated through RNAi experiments in which gene knockdown was associated with increased p27 expression and inhibition of RB protein phosphorylation (Fig. 6B). The ability of dabrafenib to inhibit CDK16 was demonstrated by the ability of the vemurafenib–CDK16 siRNA combination to mimic the effects of dabrafenib on pRB and p27 (Fig. 6B). In contrast, the combination of dabrafenib and CDK16 siRNA was not associated with any further change in either S780 RB or p27 expression. Decreased RB phosphorylation and increased p27 expression were also observed in other *NRAS*‐mutant melanoma and *KRAS*‐mutant pancreatic carcinoma cells (Fig. S10). Silencing of *CDK16* led to cell cycle arrest in the WM1366 cells, but not the 1205Lu cell line (Fig. S11). However, this was not accompanied by entry into senescence (data not shown). Combined silencing of both *CDK16* and *NEK9* also led to a significant (*P* < 0.05) reduction in the growth of both 1205Lu and WM1366 melanoma cell lines (Fig. 6C).

## 4 Discussion

Vemurafenib and dabrafenib are widely used in clinical practice for the treatment of *BRAF*‐mutant melanoma (Chapman *et al*., 2011; Larkin *et al*., 2014; Robert *et al*., 2015). Although their efficacy is similar, significant differences have been observed in their side‐effect profiles (Johnson *et al*., 2015). This led us to hypothesize that dabrafenib may inhibit a different series of kinase targets compared to vemurafenib, which could have activity against *BRAF* wild‐type tumors. In agreement with this, our initial studies showed dabrafenib to be more effective at suppressing the growth of *NRAS‐* and *KRAS*‐mutant cancer cell lines than vemurafenib.

To better understand the differences in effects between dabrafenib and vemurafenib, we undertook a chemical proteomic screen to define the spectrum of unique off‐target kinases. Chemical proteomics is an innovative method that uses the drug of interest to ‘pull down’ interacting proteins and kinases, which are then identified using mass spectrometry (Li *et al*., 2010). It offers the advantages of being unbiased and highly sensitive, as well as capturing the effects of drugs on protein–protein complexes—which cannot be achieved through *in vitro* kinase assays. Previous studies, using cell‐type agnostic *in vitro* kinase assays, have already identified other potential targets of vemurafenib (CRAF, BRAF, SRMS, MAPK4K5, FGR: IC50 all <100 nm) and dabrafenib (BRAF, CRAF, SIK2, ALK5: IC50 all <100 nm) (Bollag *et al*., 2010; Rheault *et al*., 2013). Among the three drugs tested here, trametinib had the narrowest target profile, essentially only interacting with members of the MAP2K family of kinases. This is likely to be a reflection of the highly specific nature of the allosteric MEK inhibitors compared to the ATP‐competitive BRAF inhibitors. Our chemical proteomic analyses identified a previously unidentified series of dabrafenib targets including CAMK1α, CDK16, and NEK9. NEK9 and CDK16 were chosen for further study based upon their potent inhibition by dabrafenib in *in vitro* kinase assays, and by preliminary siRNA experiments in which their silencing inhibited the growth of *NRAS*‐mutant melanoma cells.

NEK9 is part of the 11‐family member never in mitosis gene A (NIMA)‐related kinase (NEKs) serine/threonine kinases, whose primary role is in cell cycle checkpoint control. Functionally, NEK9 has been best characterized in mitosis where it helps to regulate centrosome separation and spindle assembly through the downstream kinases NEK6 and NEK7 (Belham *et al*., 2003). Expression of NEK9 increases in response to replication stress, leading to its association with CHK1 and an increase in CHK1 kinase activity (Smith *et al*., 2014). In triple‐negative breast cancer cells, silencing of *NEK9* increases levels of replication stress, leading to spontaneous DNA damage and enhanced sensitivity to DNA‐damaging chemotherapy agents (Smith *et al*., 2014). In other systems, knockdown of NEK9 expression disrupts microtubule dynamics and is associated with mitotic catastrophe (Kaneta and Ullrich, 2013). A recent unbiased proteomic screen also demonstrated NEK9 to be a potential autophagy mediator as part of the ATG8 subnetwork (Behrends *et al*., 2010). NEK9 has been implicated in several cancers with increased expression being observed in recurrent glioma and imatinib‐resistant chronic myeloid leukemia (CML) (Cooper *et al*., 2013; Varghese *et al*., 2016). It was also recently identified as a critical cell cycle regulator in cancer cell lines with inactivated p53 and was proposed as a potential therapeutic target in these tumors (Kurioka *et al*., 2014). The potential role of NEK9 in melanoma has not been previously explored.

We confirmed NEK9 to be a potent target of dabrafenib by *in vitro* kinase assays, with inhibition of NEK9 observed in the single‐digit nanomolar range. In cell‐based studies of *NRAS‐* and *KRAS‐*mutant cancer cells, dabrafenib inhibited the NEK9 target CHK1, whereas vemurafenib did not. The effects of *NEK9* knockdown manifested at the level of the cell cycle, with G1‐phase arrest, the induction of senescence markers, and increased p21 expression being observed. Although NEK9 has been primarily associated with mitosis regulation, we did not observe any mitotic catastrophe in the melanoma cell lines following its silencing. No effects were also seen with regard to NEK9 inhibition and arrest of the *NRAS*‐ or *KRAS*‐mutant cells at the G2/M spindle checkpoint. Recent work has suggested that NEK9 may also have other less‐defined roles in gene transcription and may function as a transcriptional suppressor. This has been best characterized in cells that are infected with adenovirus where NEK9 is co‐opted to suppress p53‐mediated GADD45A expression, preventing cell cycle arrest and cellular stress (Jung *et al*., 2015). More comprehensive, RNA‐Seq‐based analyses have demonstrated a role for NEK9 in the regulation of cell cycle‐related mRNAs, with its silencing leading to increased p21 expression and decreased p38 MAPK and CDK4 expression (Kurioka *et al*., 2014). Our results support these findings and reveal a role for NEK9 in controlling the expression of p21 and CDK4, with alterations of both proteins likely to explain the underlying cell cycle arrest we observed. Although our data demonstrated NEK9 to regulate CDK4 expression at the transcriptional level, the exact mechanism underlying this effect remains to be determined. A link between the cell cycle effects of *NEK9* silencing and CDK4 suppression was suggested by pharmacological studies in which the CDK4 inhibitors palbociclib and ribociclib also induced a G1‐phase cell cycle arrest and senescence in the *NRAS‐* and *KRAS*‐mutant cancer cell lines.

Recent studies have suggested that cancer cells lacking p53 function are dependent upon NEK9 for their proliferation (Kurioka *et al*., 2014). An analysis of the genetic profile of our *NRAS‐* and *KRAS‐*mutant cell lines showed many of these to harbor inactivating p53 mutations. To further determine the p53 dependency of NEK9 inhibition following dabrafenib treatment, we evaluated the effects of both of the BRAF inhibitors upon isogenic p53+/+ and p53−/− HCT116 cells and found that dabrafenib selectively inhibited pCHK1 and increased p21 expression in the cells that were p53‐null. A role for NEK9 in the growth of cancer cells lacking p53 function was further suggested by the enhanced growth inhibitory effects of dabrafenib seen in the p53−/− HCT116 cells compared to the p53+/+. Although our analysis of the melanoma TCGA database revealed high levels of NEK9 expression to be associated with a worse overall survival, we were not able to correlate this with p53 mutational status. This may be due to the fact that melanomas do not frequently harbor p53 mutations and instead inactivate their p53 through other means, such as high expression of MDM2 and MDM4 (Francoz *et al*., 2006; Gembarska *et al*., 2012; Polsky *et al*., 2001).

As our data suggested dabrafenib to be a potent inhibitor of NEK9, which also potentially hit other targets, we next turned our attention to CDK16 (or PCTAIRE1) (Mikolcevic *et al*., 2012). Multiple studies have demonstrated a role for CDK16 in cancer progression, with prostate, breast, and cervical cancer cell lines showing increased expression relative to normal cells. In cancer cells, CDK16 is involved in cell cycle regulation through its interaction with p27^KIP1^, where it catalyzes p27 phosphorylation at S10, resulting in its degradation (Yanagi *et al*., 2014a). CDK16 has also been suggested as a therapeutic target in *BRAF*‐mutant melanoma with increased expression of the protein reported in melanoma metastases compared to benign nevi (Yanagi *et al*., 2014b). Silencing of CDK16 through shRNA inhibited the growth of *BRAF*‐mutant melanoma cell lines *in vitro* and *in vivo (*Yanagi *et al*., 2014b *)*. We confirmed these findings and showed through analysis of the TCGA melanoma dataset that high expression of CDK16 was associated with a shorter overall survival. Silencing of CDK16 in both *BRAF‐* and *NRAS*‐mutant melanoma cell lines led to cell cycle arrest associated with expression of p27 and reduced phosphorylation of the RB protein at S780. In these cases, the effects of CDK16 silencing in the *RAS*‐mutant cancer cell lines were recapitulated through dabrafenib, but not vemurafenib, treatment.

Many effective anticancer drugs simultaneously inhibit multiple cellular targets (Knezevic *et al*., 2016; Wright *et al*., 2017). This phenomenon, termed polypharmacology, is probably common to many FDA‐approved drugs and its further study offers the potential to repurpose agents that have already undergone clinical safety testing. The findings contained herein reveal that a number of off‐target effects of dabrafenib, such as NEK9 and CDK16, are inhibited at clinically achievable doses and that this likely contributes to its efficacy, particularly in cancers that lack *BRAF* mutations. Significantly, we identified dabrafenib, an FDA‐approved BRAF kinase inhibitor as the first known inhibitor of NEK9. This is significant considering growing interest in NEK9 as a therapeutic target in p53‐mutant cancers including *KRAS*‐mutant colon cancer and non‐small‐cell lung cancer (NSCLC) (Kurioka *et al*., 2014). NEK9 is also highly expressed in glioblastoma, with the potential to sensitize these tumors to chemotherapy by increasing sensitivity to DNA damage (Varghese *et al*., 2016). Further studies have shown a role for NEK9 in viral replication. We therefore propose that dabrafenib could be repurposed particularly as an inhibitor of NEK9, potentially widening its clinical use into other settings.

## 5 Conclusions

We have shown for the first time that dabrafenib potently inhibits kinases not affected by vemurafenib, including NEK9 and CDK16. It is likely that NEK9 and CDK16 inhibition may contribute to the activity of dabrafenib, perhaps suggesting utility of this drug in other, non‐*BRAF*‐mutant cancers.

## Acknowledgements

This work was supported by R01 CA161107‐01, R21 CA198550, and P50 CA168536 to KSMS, R01 CA181746 to UR, and the Cancer Center Support Grant P30 CA076292 from the National Institutes of Health (NCI/NIH). IS was supported by a Career Development Award from the Melanoma Research Foundation.

## Authors contributions

MP, LR, and IS performed the experiments. BJS and YAC analyzed the TCGA data. HL performed the chemical synthesis. UR and KSMS conceived the project and wrote the manuscript. All authors read and approved the manuscript.

## Ethics approval

No animals or human specimens were used in this study.

## Availability of data and materials

The proteomic datasets analyzed are available from the corresponding authors upon request.

## Supporting information


**Fig. S1** Identification of the equipotent concentrations of the BRAF inhibitors vemurafenib and dabrafenib. 1205Lu melanoma cells were treated with increasing concentrations of each drug for 5 hrs. Western Blot shows pERK and total protein loading (GAPDH).
**Fig. S2A** Structures of vemurafenib, dabrafenib and the chemically modified form of each compound (i‐vemurafenib and i‐dabrafenib).
**Fig. S2B** Chemical structure of the MEK inhibitor trametinib and the chemically modified form i‐trametinib.
**Fig. S3** (A) The activity of vemurafenib, dabrafenib and their immobilizable analogues against BRAF V600E kinase activity. (B) The activity of trametinib and i‐trametinib against MEK2 kinase activity. Concentrations are in nm.
**Fig. S4** Chemical proteomics pulldown of 1205Lu lysates using i‐trametinib. Kinome tree shows interacting kinases of trametinib. Values given are normalized abundance spectral factors (NSAF). Lower panel: Trametinib binds MEK1/2 in 1205Lu lysates. Immobilized ampicillin is used as negative control.
**Fig. S5** siRNA knockdown of NEK9 reduces the growth of NRAS‐mutant melanoma cell lines. Cells were transfected with siRNA # 1 (Sigma) (50 nm) overnight before quantification of cell numbers by Trypan blue.
**Fig. S6** Knockdown of Nek9 does not induce apoptosis in 1205Lu and WM1366 melanoma cell lines. Cells were transfected with Nek9 siRNA # 1 (Sigma) (50 nm) overnight. Cells were then stained for Annexin V.
**Fig. S7** Nek9 silencing with siRNA # 2 (Dharmacon) leads to G0/G1 phase cell cycle arrest in 1205Lu and WM1366 cells.
**Fig. S8** The CDK4 inhibitors palbociclib and ribociclib induce senescence in CAPAN‐1 and Mia PACA‐2 pancreatic cancer cell lines. Cells were treated for 5 days with drug before being stained for β‐galactosidase.
**Fig. S9** The CHK1 inhibitor SCH900776 does not induce cell cycle arrest or senescence in 1205Lu or WM1366 melanoma cells. (left) Cells were treated with drug (300 nm) for 24 hrs before being stained with propidium iodide and analyzed by flow cytometry. (right) WM1366 cells were treated for 5 days with drug before being stained for β‐galactosidase.
**Fig. S10** Western blot of pRB (S780) and p27 in IPC‐298 (NRAS‐mutant melanoma), M245 (NRAS‐mutant melanoma), Mia PACA‐2 (KRAS‐mutant pancreatic) and CAPAN‐1 (KRAS‐mutant pancreatic) cells following knockdown of CDK16.
**Fig. S11** The cell cycle effects of CDK16 knockdown in 1205Lu and WM1366 cells. Silencing of CDK16 leads to a slight G1‐phase arrest in the NRAS‐mutant WM1366 cells. Cells were treated with siRNA overnight, allowed to recover for 48 h, stained with propidium iodide and analyzed by flow cytometry.
**Table S1** Mutational profiles of the cell lines used in this study.

**Appendix S1** Synthesis of i‐vemurafenib (YL9‐155).
