# Supplementary material for: Dabrafenib inhibits the growth of BRAF‐WT cancers through CDK16 and NEK9 inhibition
Source: Mol Oncol. 2017 Nov 23;12(1):74–88. doi: 10.1002/1878-0261.12152 (PMC5748485; doi:10.1002/1878-0261.12152)
Supplement: Supplementary file 1 — Fig. S1 Identification of the equipotent concentrations of the BRAF inhibitors vemurafenib and dabrafenib. 1205Lu melanoma cells were treated with increasing concentrations of each drug for 5 hrs. Western Blot shows pERK and total protein loading (GAPDH). Fig. S2A Structures of vemurafenib, dabrafenib and the chemically modified form of each compound (i‐vemurafenib and i‐dabrafenib). Fig. S2B Chemical structure of the MEK inhibitor trametinib and the chemically modified form i‐trametinib. Fig. S3 (A) The activity of vemurafenib, dabrafenib and their immobilizable analogues against BRAF V600E kinase activity. (B) The activity of trametinib and i‐trametinib against MEK2 kinase activity. Concentrations are in nm. Fig. S4 Chemical proteomics pulldown of 1205Lu lysates using i‐trametinib. Kinome tree shows interacting kinases of trametinib. Values given are normalized abundance spectral factors (NSAF). Lower panel: Trametinib binds MEK1/2 in 1205Lu lysates. Immobilized ampicillin is used as negative control. Fig. S5 siRNA knockdown of NEK9 reduces the growth of NRAS‐mutant melanoma cell lines. Cells were transfected with siRNA # 1 (Sigma) (50 nm) overnight before quantification of cell numbers by Trypan blue. Fig. S6 Knockdown of Nek9 does not induce apoptosis in 1205Lu and WM1366 melanoma cell lines. Cells were transfected with Nek9 siRNA # 1 (Sigma) (50 nm) overnight. Cells were then stained for Annexin V. Fig. S7 Nek9 silencing with siRNA # 2 (Dharmacon) leads to G0/G1 phase cell cycle arrest in 1205Lu and WM1366 cells. Fig. S8 The CDK4 inhibitors palbociclib and ribociclib induce senescence in CAPAN‐1 and Mia PACA‐2 pancreatic cancer cell lines. Cells were treated for 5 days with drug before being stained for β‐galactosidase. Fig. S9 The CHK1 inhibitor SCH900776 does not induce cell cycle arrest or senescence in 1205Lu or WM1366 melanoma cells. (left) Cells were treated with drug (300 nm) for 24 hrs before being stained with propidium iodide and analyzed by flow cyto [file MOL2-12-74-s001.docx]

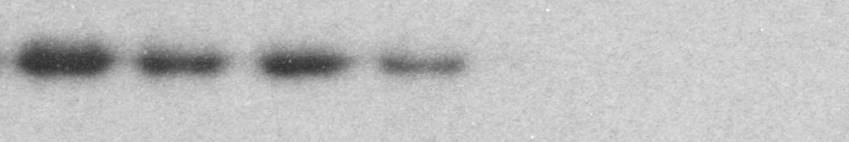

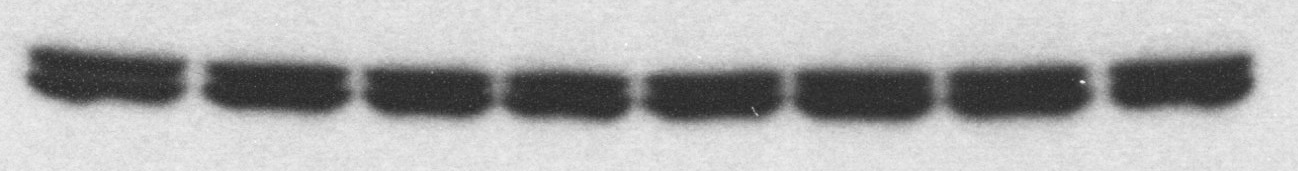

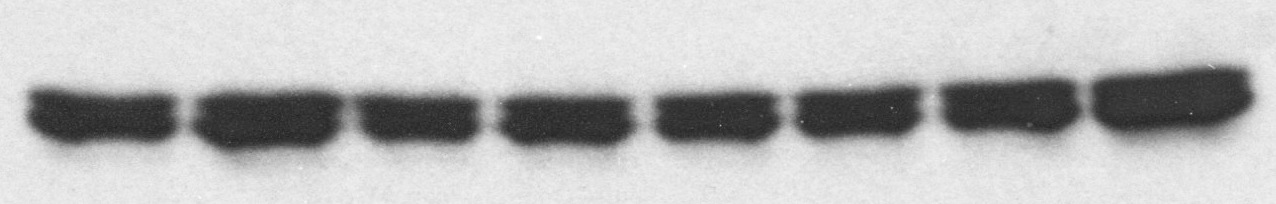


CT

10 nM

30 nM

100 nM

300 nM

1 μM

3 μM

10 μM

CT

10 nM

30 nM

100 nM

300 nM

1 μM

3 μM

10 μM

pErk

Total Erk

pErk

Total Erk

**Vemurafenib**

**Dabrafenib**


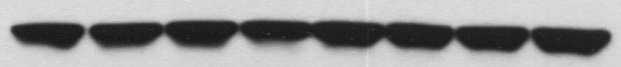


GAPDH


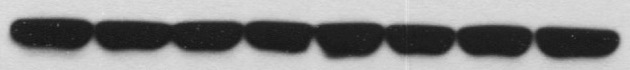


GAPDH


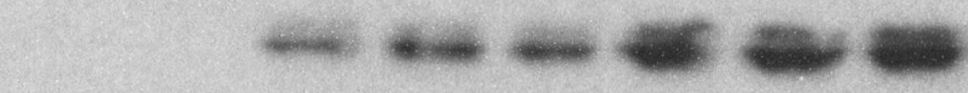


**Supplemental Figure 1:** Identification of the equipotent concentrations of the BRAF inhibitors vemurafenib and dabrafenib. 1205Lu melanoma cells were treated with increasing concentrations of each drug for 5 hrs. Western Blot shows pERK and total protein loading (GAPDH).


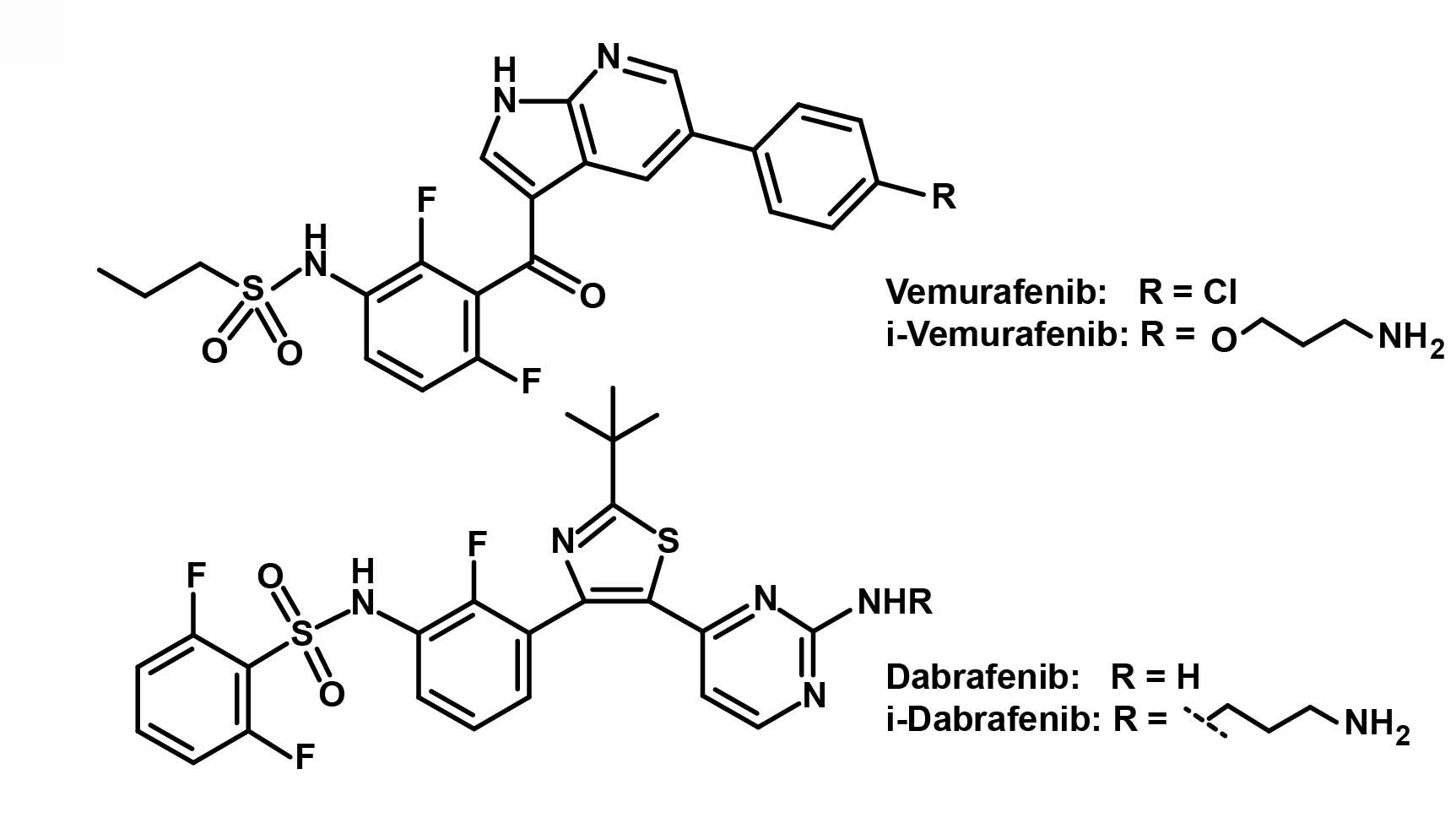


**Supplemental Figure 2A:** Structures of vemurafenib, dabrafenib and the chemically modified form of each compound (i-vemurafenib and i-dabrafenib).


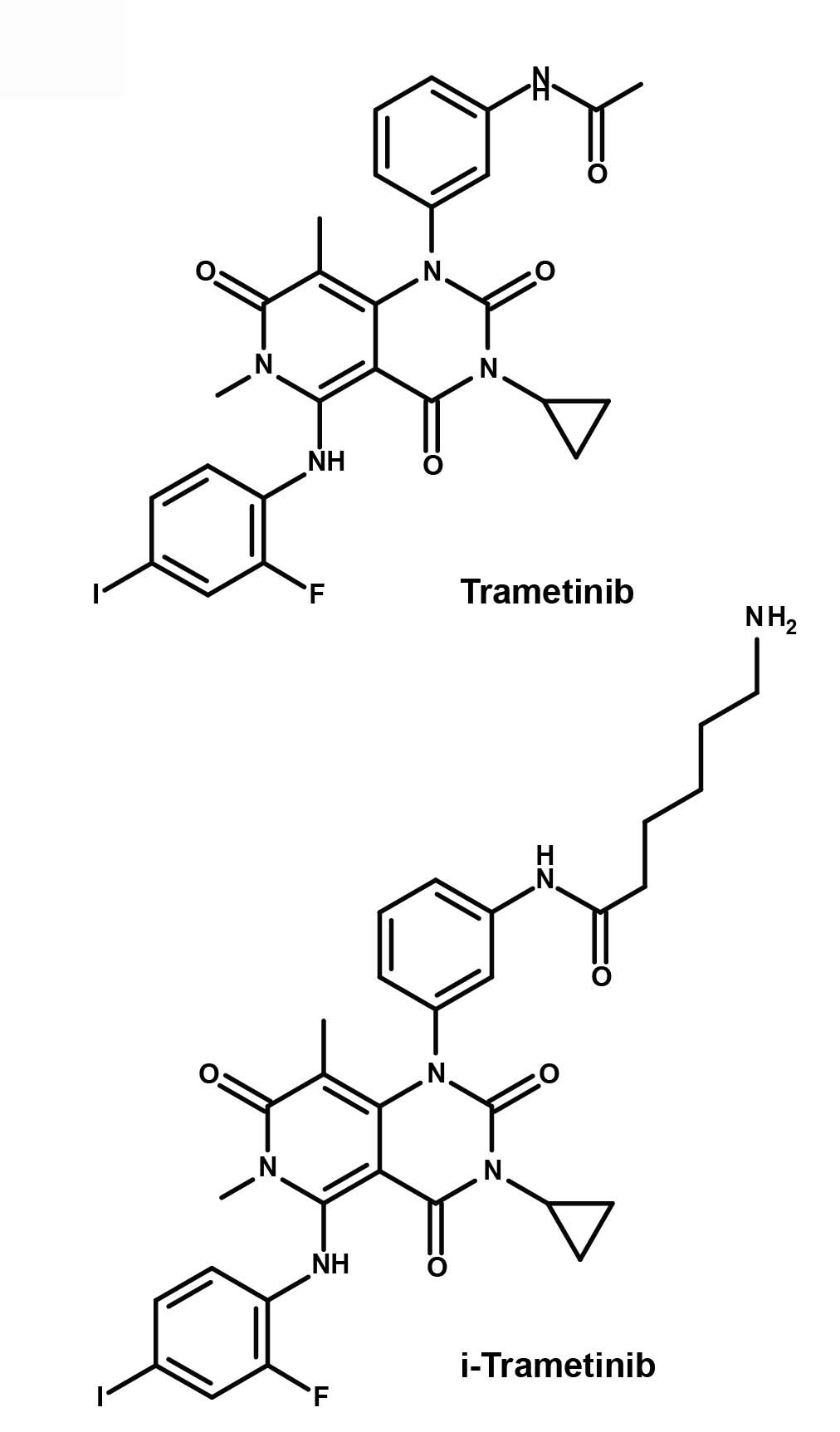


**Supplemental Figure 2B:** Chemical structure of the MEK inhibitor trametinib and the chemically modified form i-trametinib.


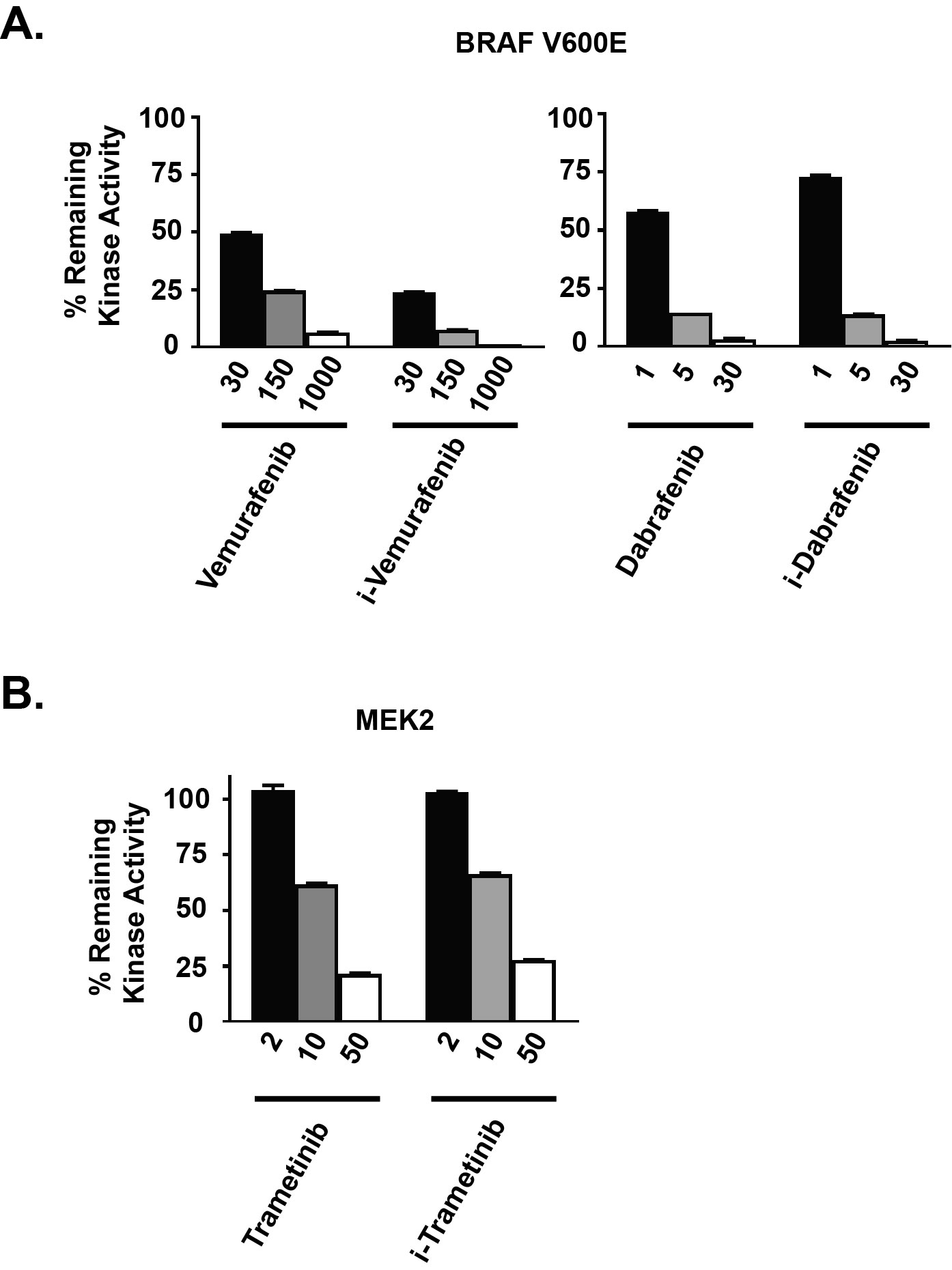


**Supplemental Figure 3: A:** The activity of vemurafenib, dabrafenib and their immobilizable analogues against *BRAF* V600E kinase activity. **B:** The activity of trametinib and i-trametinib against MEK2 kinase activity. Concentrations are in nM.


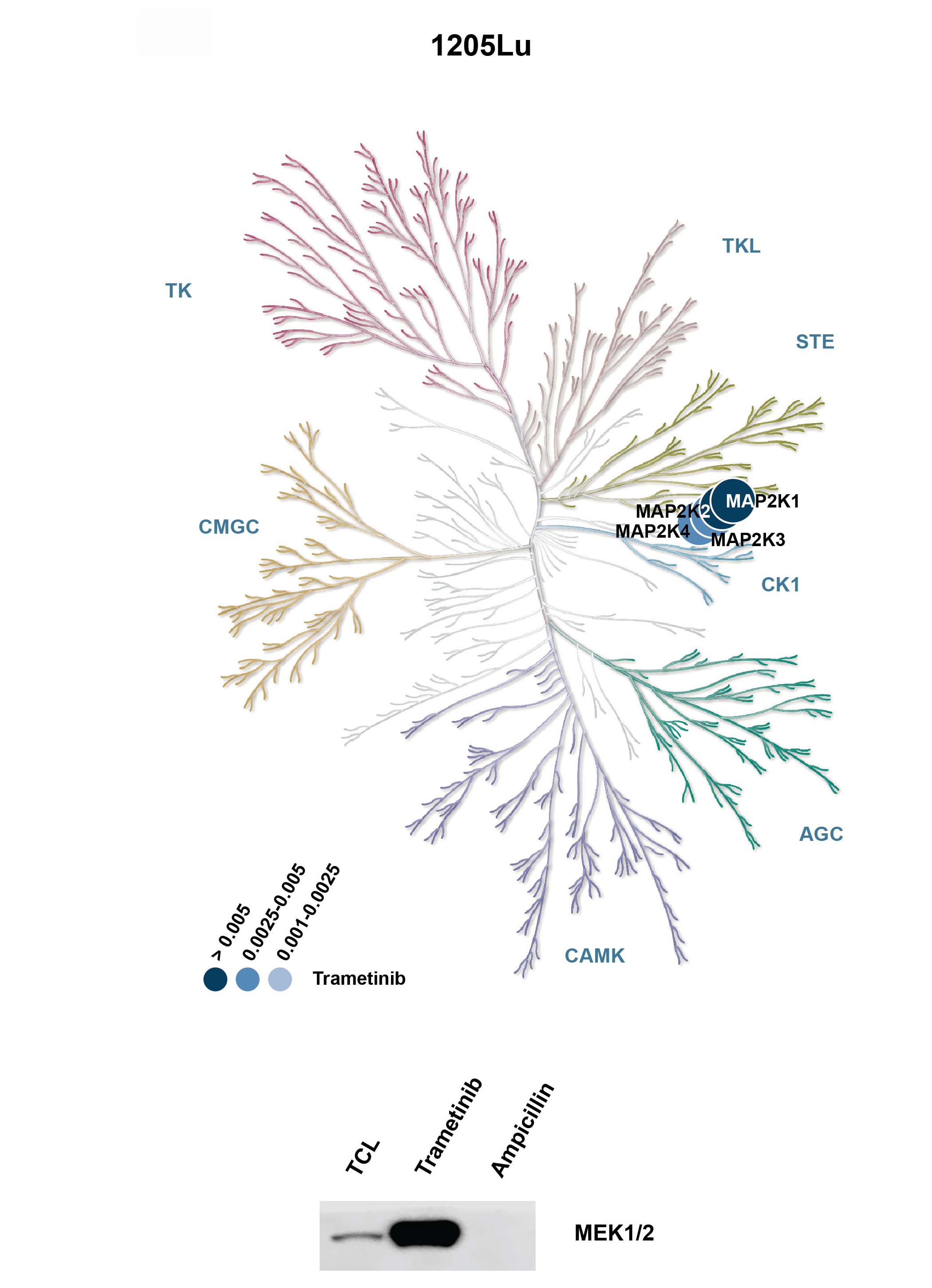


**Supplemental Figure 4:** Chemical proteomics pulldown of 1205Lu lysates using i-trametinib. Kinome tree shows interacting kinases of trametinib. Values given are normalized abundance spectral factors (NSAF). Lower panel: Trametinib binds MEK1/2 in 1205Lu lysates. Immobilized ampicillin is used as negative control.


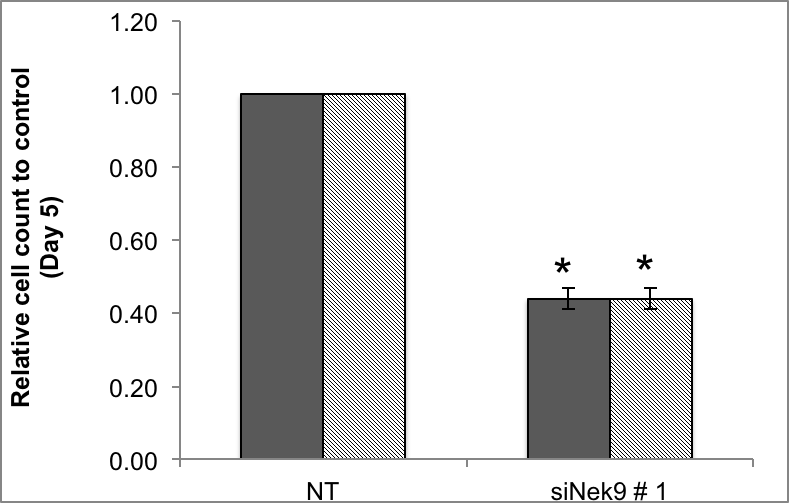


M245

IPC-298

**Supplemental Figure 5**: **siRNA knockdown of *NEK9* reduces the growth of *NRAS*-mutant melanoma cell lines.** Cells were transfected with siRNA # 1 (Sigma) (50 nM) overnight before quantification of cell numbers by Trypan blue.


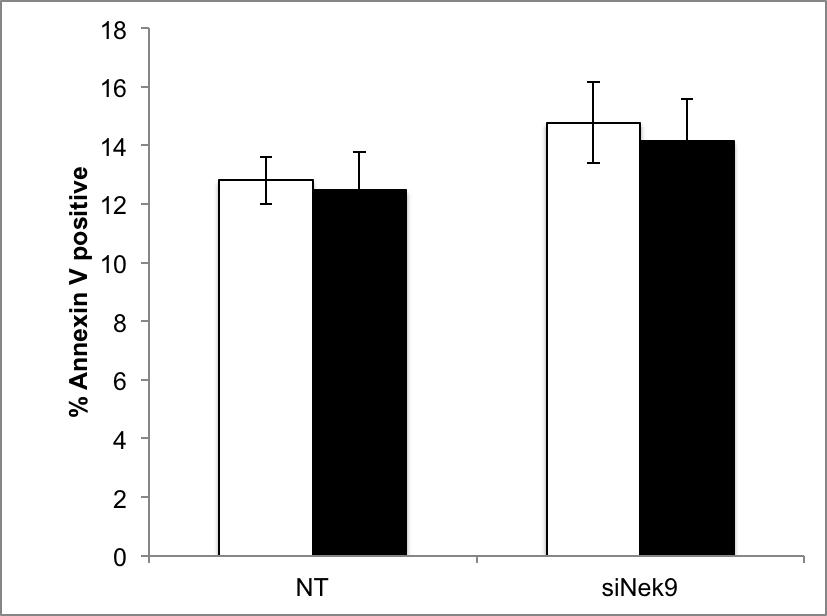


NT

siNek9 #1

WM1366

1205Lu

**Supplemental Figure 6: Knockdown of Nek9 does not induce apoptosis in 1205Lu and WM1366 melanoma cell lines.** Cells were transfected with Nek9 siRNA # 1 (Sigma) (50 nM) overnight. Cells were then stained for Annexin V.


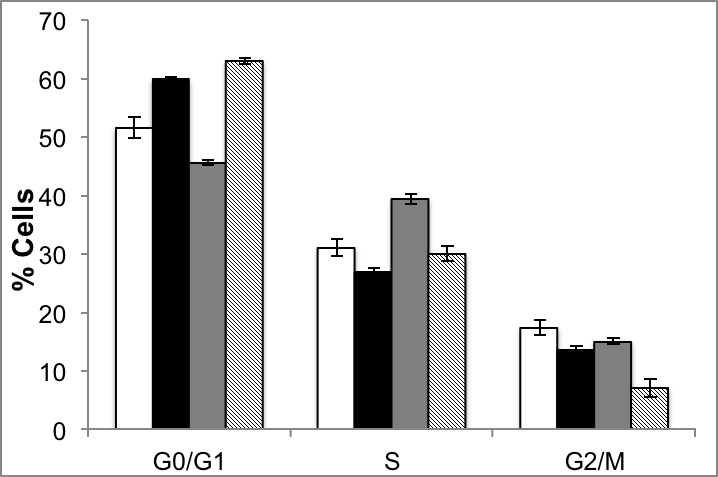


**Supplemental Figure 7**: Nek9 silencing with siRNA # 2 (Dharmacon) leads to G0/G1 phase cell cycle arrest in 1205Lu and WM1366 cells.

1205Lu NT

1205Lu siNek9 #2

WM1366 NT

WM1366 siNek9 #2


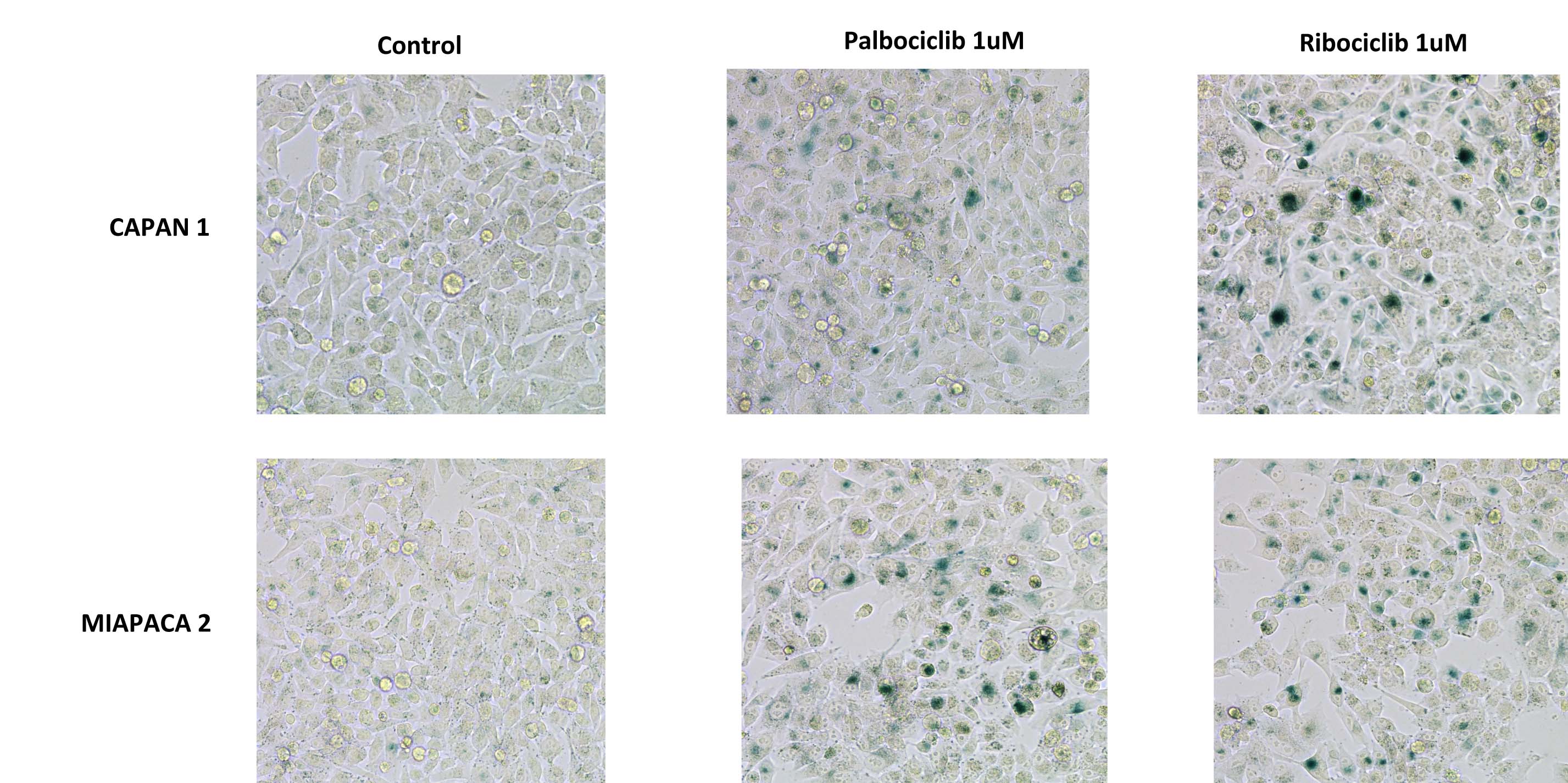


**Supplemental Figure 8:** The CDK4 inhibitors palbociclib and ribociclib induce senescence in CAPAN-1 and Mia PACA-2 pancreatic cancer cell lines. Cells were treated for 5 days with drug before being stained for β-galactosidase


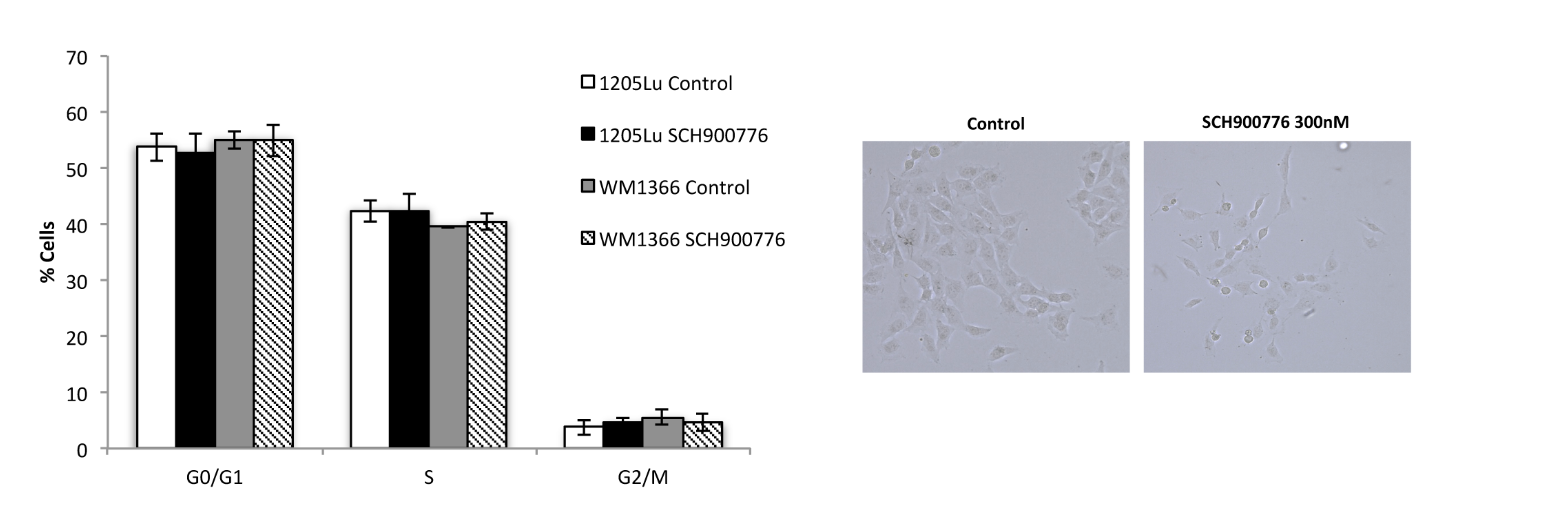


**Supplemental Figure 9:** The CHK1 inhibitor SCH900776 does not induce cell cycle arrest or senescence in 1205Lu or WM1366 melanoma cells. (left) Cells were treated with drug (300 nM) for 24 hrs before being stained with propidium iodide and analyzed by flow cytometry. (right) WM1366 cells were treated for 5 days with drug before being stained for β-galactosidase.


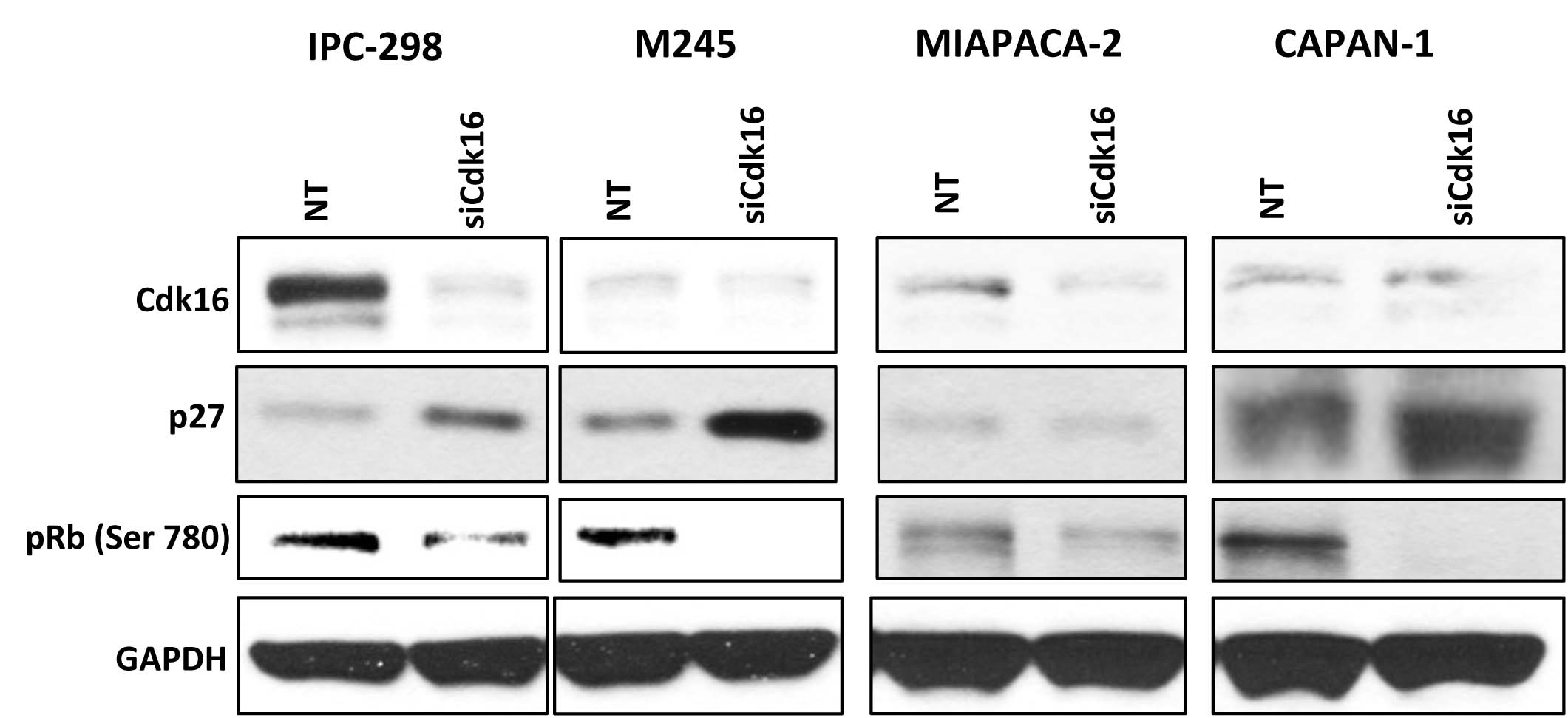


**Supplemental Figure 10:** Western blot of pRB (S780) and p27 in IPC-298 (*NRAS-*mutant melanoma), M245 (*NRAS*-mutant melanoma), Mia PACA-2 (*KRAS*-mutant pancreatic) and CAPAN-1 (*KRAS*-mutant pancreatic) cells following knockdown of CDK16.


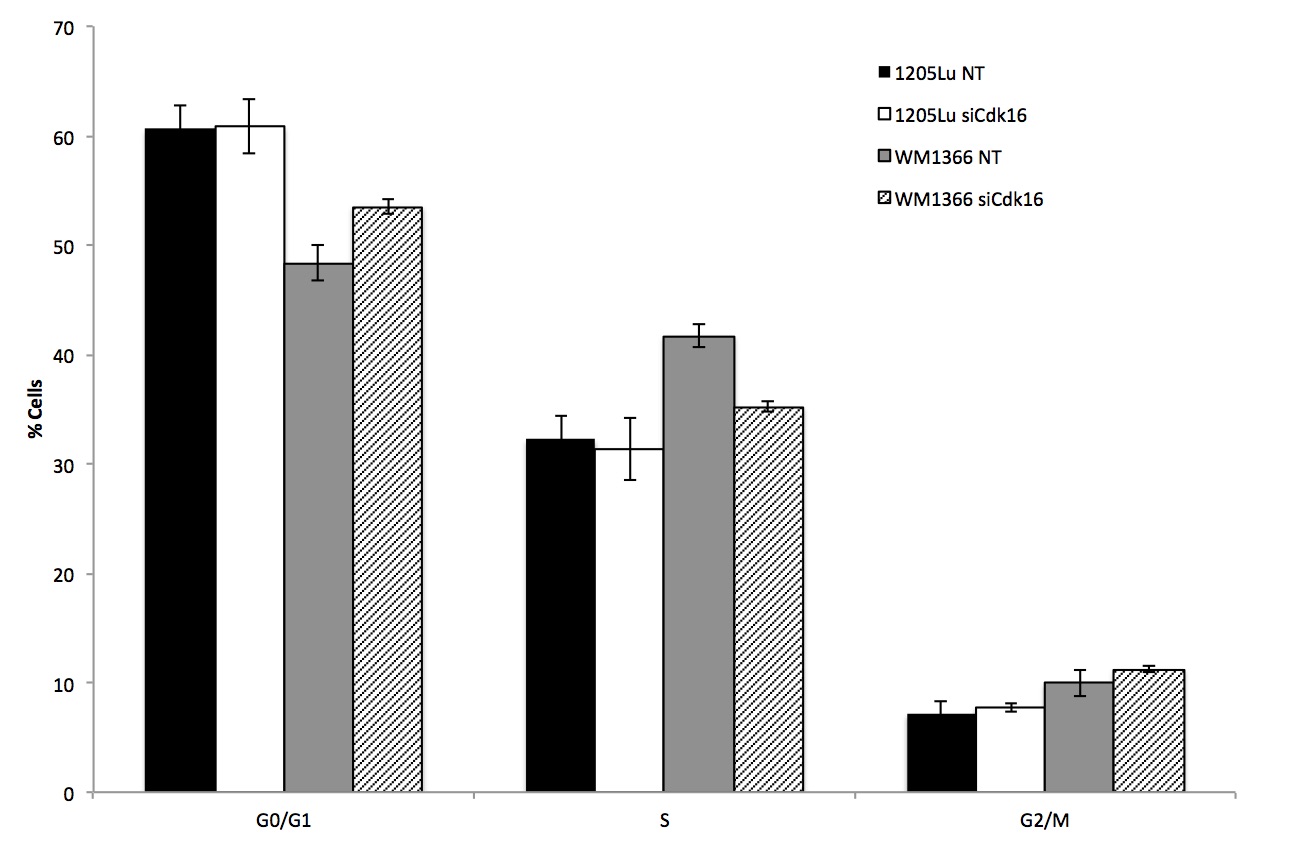


**Supplemental Figure 11:** The cell cycle effects of CDK16 knockdown in 1205Lu and WM1366 cells. Silencing of CDK16 leads to a slight G1-phase arrest in the *NRAS*-mutant WM1366 cells. Cells were treated with siRNA overnight, allowed to recover for 48 hr, stained with propidium iodide and analyzed by flow cytometry.

| Cell line | RAF/ RAS Mutation | TP53 status |
| --- | --- | --- |
| 1205Lu | BRAF V600E | Wild-type |
| WM1366 | NRAS Q61L | Mutant (E258K Ex8) |
| IPC-298 | NRAS Q61L | Mutant (R213 and homozygous deletion) |
| M245 | NRAS Q61K | Wild-type |
| MIA PACA-2 | KRAS G12C | Mutant C742T and homozygous deletion |
| CAPAN-1 | KRAS G12V | Mutant C159T |
| HCT-116 | KRAS G13D | Wild-type |

**Supplemental Table 1:** Mutational profiles of the cell lines used in this study.
