# Supplementary material for: Dabrafenib inhibits the growth of BRAF‐WT cancers through CDK16 and NEK9 inhibition
Source: Mol Oncol. 2017 Nov 23;12(1):74–88. doi: 10.1002/1878-0261.12152 (PMC5748485; doi:10.1002/1878-0261.12152)

**Supplementary Methods**

**Synthesis of i-vemurafenib (YL9-155):**

**3-(4-(4,4,5,5-tetramethyl-1,3,2-dioxaborolan-2-yl)phenoxy)propylcarbamate intermediate (YL9-150)**: A mixture of 4-hydroxyphenylboronic acid pinacol ester (0.440 g, 2.0 mmol), *tert*-butyl 3-bromopropylcarbamate (0.714 g, 3 mmol), Cs_2_CO_3_ (1.060 g, 3 mmol), KI (0.033 g, 0.1 mmol) and tetrabutylammonium iodide (0.074 g, 0.2 mmol) in anhydrous DMF (10 mL) was heated in a sealed tube at 60 ºC for 18 h. The mixture was poured into water (25 mL) upon cooling and extracted with EtOAc (50 mL). The organic phase was washed with water (25 mL x 4), brine (25 mL), then dried over Na_2_SO_4_, filtered and concentrated to dryness. The material was purified by SiO2 flash chromatography (10 g column, 0-15% EtOAC in hexane) to afford the title compound white solid (0.665 g, 88%), as the major product. This intermediate was used in the next step without further purification. ^1^H NMR (400 MHz, CDCl_3_): δ 7.22 (d, *J* = 9.2 Hz, 2H) 6.81 (d, *J* = 9.2 Hz, 2H) 3.98 (t, *J* = 6.0 Hz, 2H) 3.34-3.30 (m, 2H), 1.97 (t, *J* = 6.0 Hz, 3H), 1.44 (s, 12H), 1.27 (s, 9H); HPLC-MS (ESI+) *m/z* 400.4 (M+ Na)^+^, 777.6 (2M + Na)^+^.

***N*-(3-(5-(4-(3-aminopropoxy)phenyl)-1H-pyrrolo[2,3-b]pyridine-3-carbonyl)-2,4-difluorophenyl)propane-1-sulfonamide bis TFA salt (YL9-155)**: The **YL9-146** was synthesized as reported in the literature (**Ref. WO 2012/010538 A2; Novel process for the manufacture of *N*-(3-(5-(4-chlorophenyl)-1H-pyrrolo[2,3-b]pyridine-3-carbonyl)-2,4-difluorophenyl)propane-1-sulfonamide**) A mixture of **YL9-146** (0.092 g, 0.2 mmol), **YL9-150** (0.108 g, 0.286 mmol) and aqueous K_2_CO_3_ (1M solution, 2.86 mL, 2.86 mmol) in acetonitrile (5.7 mL) was degassed using argon for 10 min., and added (PPh_3_)_4_Pd (0.023 g, 0.02 mmol) under argon atmosphere. The reaction mixture was then degassed again for additional 5 min. The sealed tube was heated at 100 ºC for 18 h. The reaction mixture was poured into a separation funnel containing water (30 mL) upon cooling and extracted with ether (30 mL x2). The combined ether layer was washed with brine (20 mL), dried over Na_2_SO_4_, filtered and concentrated to dryness. The crude material was purified by SiO2 flash chromatography (EtOAc/Hexane gradient elution. The compound obtained was recrystallized with EtOAC/Hex (1:2) to afford the title compound as a beige color solid (0.084 g, 67%). HPLC 98.0% (*t*_R_ = 10.48 min, 50% MeOH in 0.1% TFA water 20 min); ^1^H NMR (400 MHz, DMSO-*d_6_*): δ 12.99 (s, 1H disappeared on D_2_O shake), 9.78 (s, 1H disappeared on D_2_O shake), 8.65 (appd, *J* = 2.0 Hz, 1H), 8.57 (s, 1H), 8.21 (s, 1H), 7.84 (brs, 3H disappeared on D_2_O shake), 7.68 (d, *J* = 8.8 Hz, 2H), 7.60-7.54 (m, 1H), 7.27 (t, *J* = 8.8 Hz, 1H), 7.08 (d, *J* = 8.8 Hz, 2H), 4.12 (t, *J* = 6.0 Hz, 2H), 3.11 (t, *J* = 8.0 Hz, 2H), 3.01-2.97 (m, 2H), 2.06-2.00 (m, 2H), 1.77-1.67 (m, 2H), 0.94 (t, *J* = 7.6 Hz, 3H); ^19^F NMR (376 MHz, DMSO-*d_6_*): δ -74.28 (s, 6F), -117.24 (s, 1F), -122.55 (d, *J* = 7.5 Hz, 1F); HPLC-MS (ESI+) *m/z* 529.16 (M + Na)^+^; HRMS (ESI +) *m/z* calculated for C_26_H_27_F_2_N_4_O_4_S (M + H)^+^ 529.1716, found 529.1707.

**Synthesis of i-dabrafenib (YL10-041):**

***Tert*-butyl (3-((4-(2-(tert-butyl)-4-(3-((2,6-difluorophenyl)sulfonamido)-2-fluorophenyl)thiazol-5-yl)pyrimidin-2-yl)amino)propyl)carbamate bis TFA salt (YL10-041)**: The **YL10-31** was synthesized as reported in the literature (**Ref. WO 2012/010538 A2; Novel process for the manufacture of *N*-(3-(5-(4-chlorophenyl)-1H-pyrrolo[2,3-b]pyridine-3-carbonyl)-2,4-difluorophenyl)propane-1-sulfonamide**). The **YL10-031** (200 mg, 0.371 mmol), *tert*-butyl (3-aminopropyl)carbamate (388 mg, 2.22 mmol) in THF (0.5 mL) were heated at 75 ºC in a sealed tube for 16 h. The THF was evaporated, and the mixture was dissolved in DCM (25 mL), washed with HCl (1M, 20 mL), and the aqueous layer was extracted with DCM (10 x 2 mL). The combined DCM layer was washed with brine (20 mL), dried over Na_2_SO_4_, filtered and concentrated. The crude material was purified by flash chromatography (SiO_2_, EtOAc-Hexane gradient elution) to afford a white solid (202 mg, 80%) as the Boc protected intermediate. The BOC protected precursor of **YL10-041** (154 mg, 2 mmol) was dissolved in DCM (3.0 mL) and TFA (3.0 mL) was added at 0 ^o^C. The reaction mixture was stirred at r.t. for 1-2 h, solvent was removed, and the resulting mixture was slurried in DCM and hexane. The white solid was filtered and washed with ether (3 ml x 2), and dried to afford pure **YL10-041** TFA salt (159 mg, 87%). HPLC 91.0% (*t*_R_ = 5.76 min, 60% MeOH in 0.1% TFA water 20 min); ^1^H NMR (400 MHz, DMSO-*d_6_*): δ 10.87 (s, 1H disappeared on D_2_O shake), 8.05 (d, *J* = 4.8 Hz, 1H), 7.75 (brs, 2H, disappeared on D_2_O shake), 7.70-7.62 (m, 1H), 7.45 (brs, 1H, disappeared on D_2_O shake), 7.39 (dt, *J* = 8.0 Hz, 1.6 Hz, 1H), 7.34 (dt, *J* = 8.0 Hz, 1.6 Hz, 1H), 7.28-7.20 (m, 2H) 5.99-5.87 (brd, 1H), 3.34-3.21 (brm, 2H), 2.81 (brs, 2H), 1.75 (brs, 2H), 1.38 (s, 9H); ^19^F NMR (376 MHz, DMSO-*d_6_*): δ -74.28 (s, 6F), -107.43 (t, *J* = 7.52 Hz, 2F), -124.36 (s, 1F); HPLC-MS (ESI+) *m/z* 577.2 (M + H)^+^; HRMS (ESI +) *m/z* calculated for C_26_H_27_F_3_N_6_O_2_S_2_ (M + H)^+^ 577.1661, found 577.1670.

**Synthesis of i-trametinib (YL10-103):**

**6-Amino-*N*-(3-(3-cyclopropyl-5-((2-fluoro-4-iodophenyl)amino)-6,8-dimethyl-2,4,7-trioxo-3,4,6,7-tetrahydropyrido[4,3-d]pyrimidin-1(2H)-yl)phenyl)hexanamide TFA salt (YL10-103)**: The **YL10-097** intermediate was synthesized as reported in the literature (**WO 2005/121142, 5-Amino-2,47-trioxo-3,4,7,8-tetrahydro-2H-pyrido’2,3-dipyrimidine derivatives and related compounds for treatment of cancer**). A mixture of Boc-aminohexanoic acid (78.0 mg, 0.33 mmol), EDC (97.0 mg, 0.50 mmol), HOBt (68 mg, 0.506 mmol) and DIPEA (0.47 mL, 2.69 mmol) in DMF ( 5.0 mL) was stirred under inert conditions for 1h at r.t. The **YL10-097** (270 mg, 0.337 mmol) was added portion wise and DMSO (2-3 mL) was added as a co-solvent to to obtain a clear solution. The reaction mixture was clear with time and stirred overnight (18 h). The conversion was about 50%. (extra reaction time at r.t. or heating did not make a difference). The DMF and DMSO was removed using Biotage V-10 evaporator, the residue obtained was dissolved in EtOAc (50 mL), washed with water (20 mL), HCl (0.1M, 20 mL), saturated Na_2_CO_3_ (20 mL) and brine (20 mL). The organic layer was dried (Na_2_SO_4_) and concentrated. The crude mixture was separated by preparative HPLC (gradient acetonitrile/water 50-95%, 30 min, 20 mL/min). The required compound **YL10-103** (50 mg, 34%) was obtained by concentrating the fractions that were eluted at 16-17 min. HPLC 99% (*t*_R_ = 5.89 min, 70% MeOH in 0.1% TFA water 20 min); ^1^H NMR (400 MHz, DMSO-*d_6_*): δ 11.06 (s, 1H, disappeared on D_2_O shake), 10.09 (s, 1H, disappeared on D_2_O shake), 7.77 (dd, *J* = 10.0, 2.0 Hz, 1H), 7.72 (brs, 2H, disappeared on D_2_O shake), 7.62 (appt, *J* = 2.0 Hz, 1H), 7.59 (appdd, *J* = 7.6, 2.0 Hz, 1H), 7.53 (dd, *J* = 8.4, 2.0 Hz, 1H), 7.33 ( t, *J* = 8.0 Hz, 1H), 7.00 (dd, *J* = 7.6, 1.2 Hz, 1H), 6.90 (t, *J* = 8.4 Hz, 1H), 3.05 (s, 3H), 2.80-2.72 (m, 2H), 2.62-2.56 (m, 1H), 2.30 (t, *J* = 7.2 Hz, 2H), 1.61-1.49 (m, 4H), 1.35-1.29 (m, 2H), 1.23 (s, 3H), 0.95-0.90 (m, 2H), 0.66-0.61 (m, 2H). ^19^F NMR (376 MHz, DMSO-*d_6_*): δ -73.72 (s, 3F), -124.36 (t, *J* = 9.0 Hz, 1F); HPLC-MS (ESI+) *m/z* 687.2 (M + H)^+^; HRMS (ESI +) *m/z* calculated for C_30_H_32_FIN_6_O_4_ (M + H)^+^ 687.1586, found 687.1581.

**^1^H NMR of i-vemurafenib (YL9-155)**


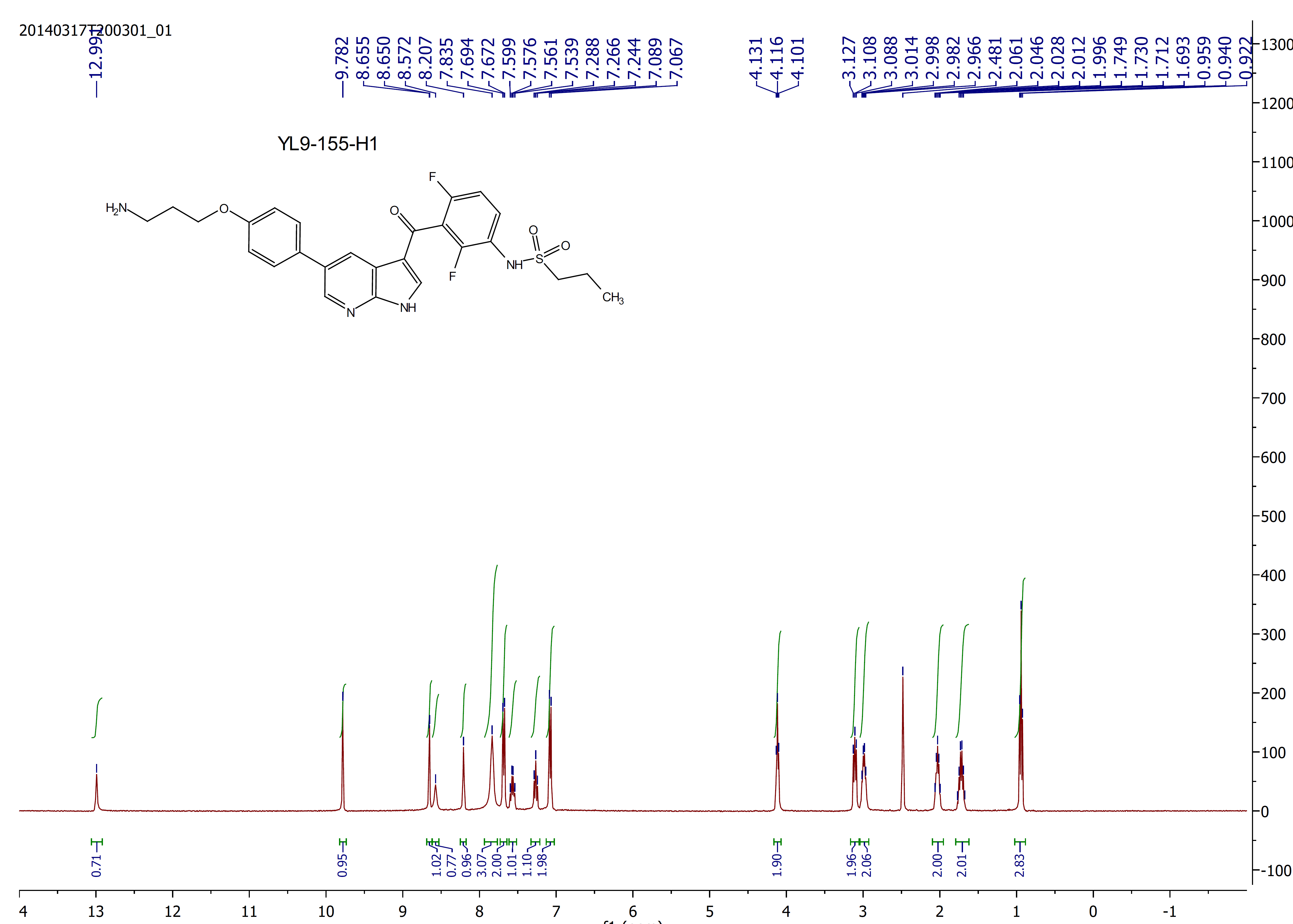


**HPLC-MS of i-vemurafenib (YL9-155)**

**^1^H NMR of i-dabrafenib (YL10-041)**


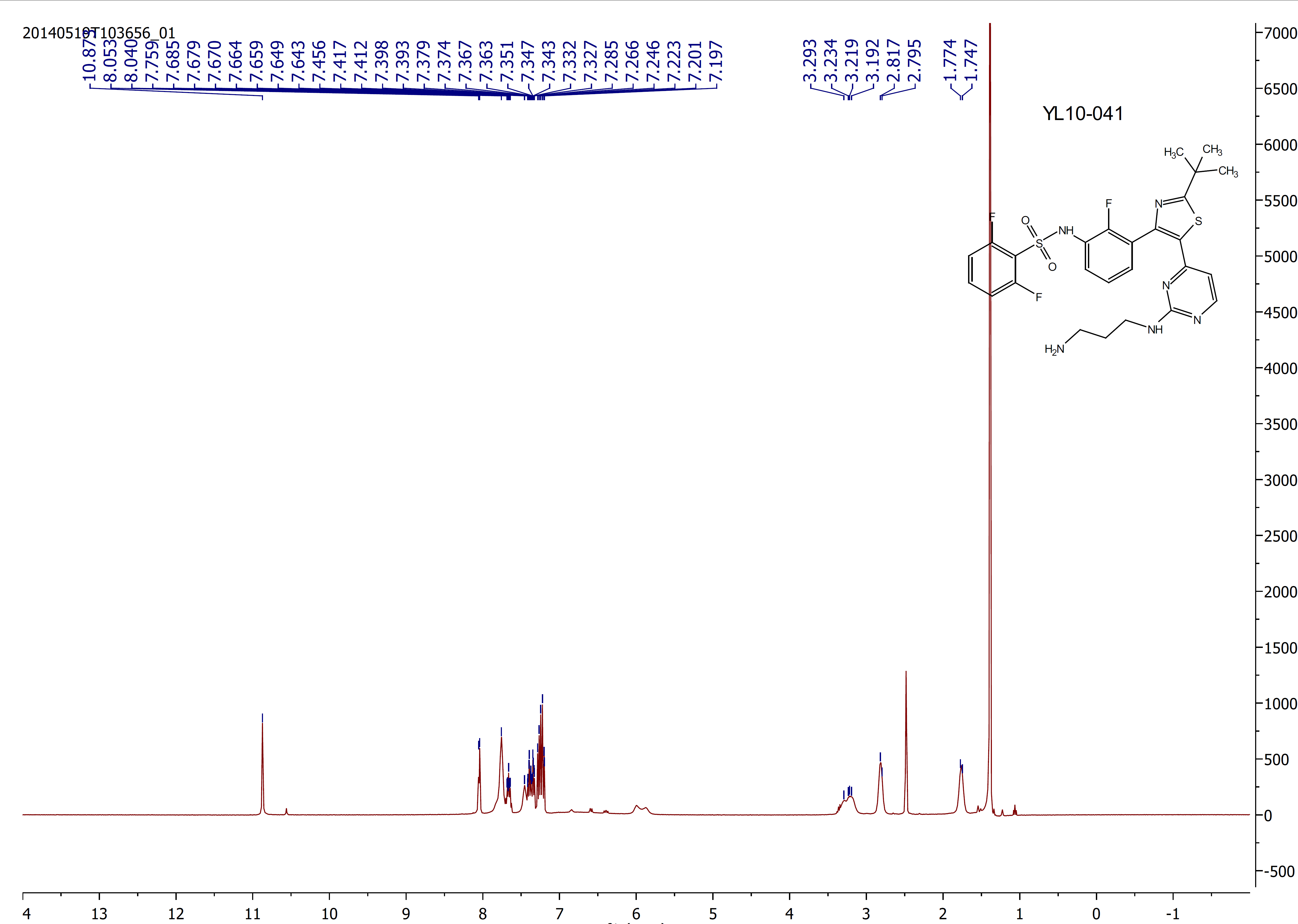


**HPLC-MS of i-dabrafenib (YL10-041)**


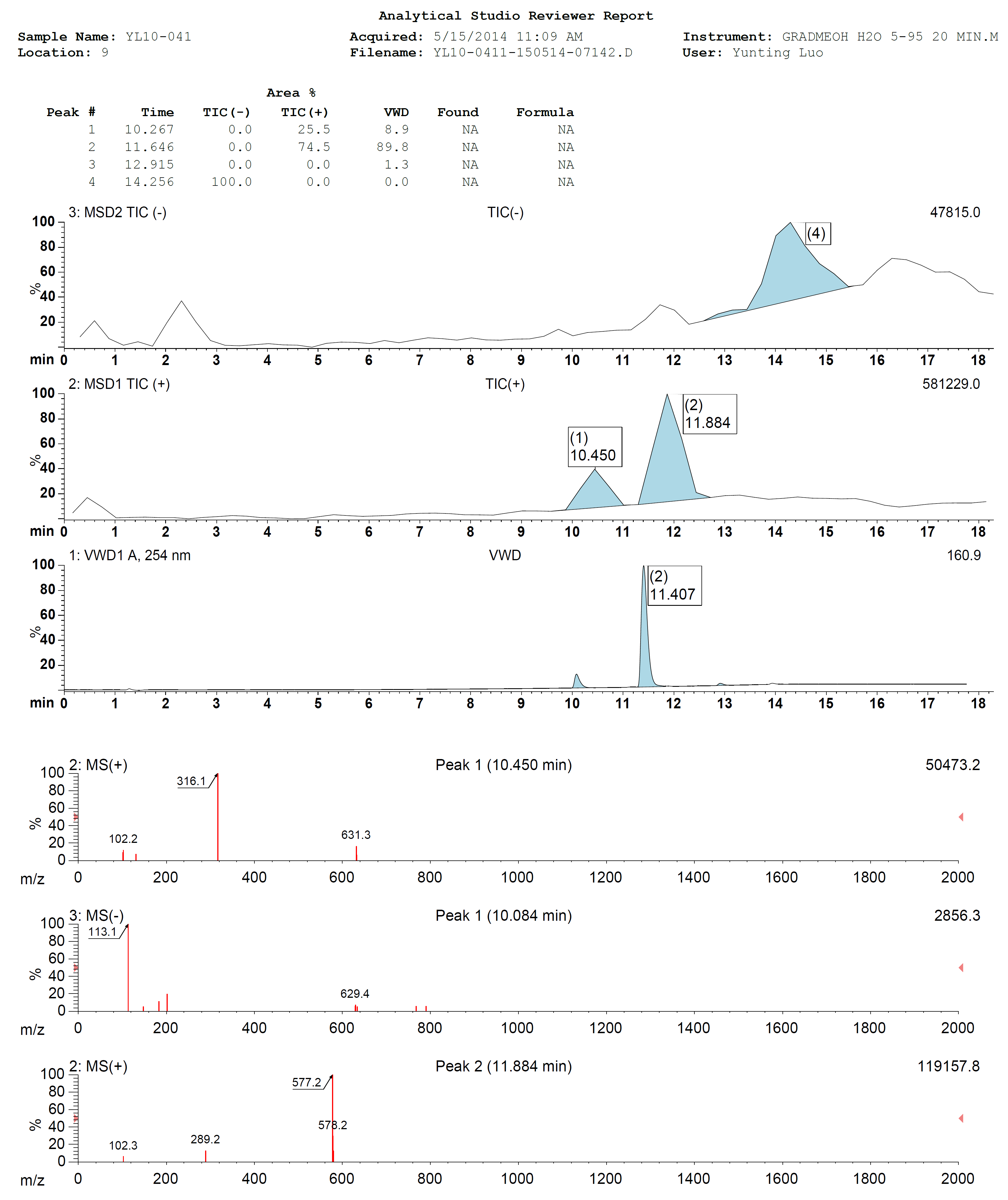


**^1^H NMR of i-trametinib (YL10-103)**


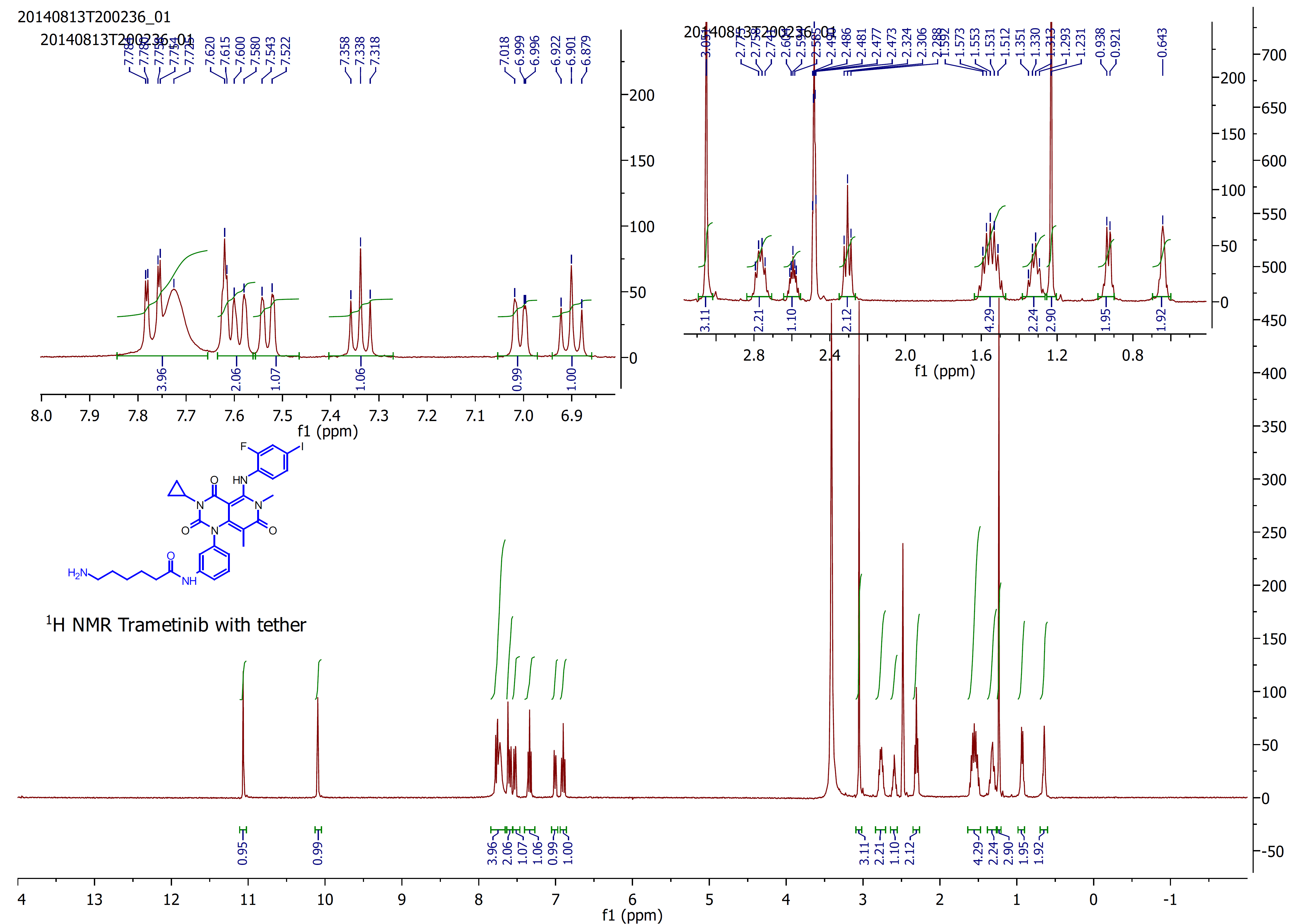


**HPLC-MS of i-trametinib (YL10-103)**


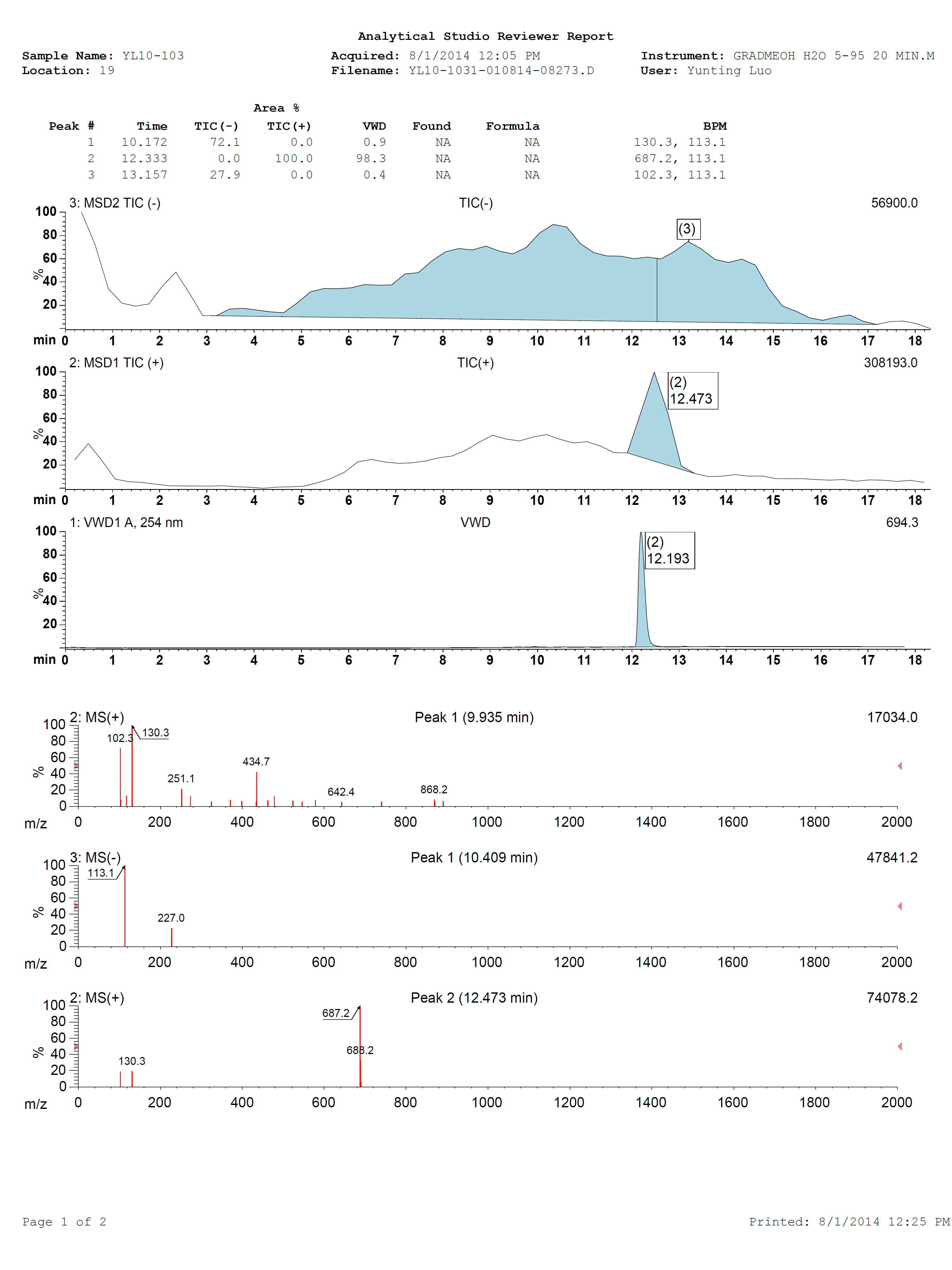

Supplement: Supplementary file 2 — Appendix S1 Synthesis of i‐vemurafenib (YL9‐155). [file MOL2-12-74-s002.doc]
